# Lipid droplet formation in *Mycobacterium tuberculosis* infected macrophages requires IFN-γ/HIF-1α signaling and supports host defense

**DOI:** 10.1371/journal.ppat.1006874

**Published:** 2018-01-25

**Authors:** Matthew Knight, Jonathan Braverman, Kaleb Asfaha, Karsten Gronert, Sarah Stanley

**Affiliations:** 1 Department of Plant and Microbial Biology, University of California, Berkeley, Berkeley, California, United States of America; 2 Koch Institute for Integrative Cancer Research, Massachusetts Institute of Technology, Cambridge, Massachusetts, United States of America; 3 Vision Science Program, School of Optometry, University of California, Berkeley, Berkeley, California, United States of America; 4 Department of Molecular and Cell Biology, Division of Immunology and Pathogenesis, University of California, Berkeley, Berkeley, California, United States of America; 5 School of Public Health, Division of Infectious Diseases and Vaccinology, University of California, Berkeley, Berkeley, California, United States of America; New Jersey Medical School, UNITED STATES

## Abstract

Lipid droplet (LD) formation occurs during infection of macrophages with numerous intracellular pathogens, including *Mycobacterium tuberculosis*. It is believed that *M*. *tuberculosis* and other bacteria specifically provoke LD formation as a pathogenic strategy in order to create a depot of host lipids for use as a carbon source to fuel intracellular growth. Here we show that LD formation is not a bacterially driven process during *M*. *tuberculosis* infection, but rather occurs as a result of immune activation of macrophages as part of a host defense mechanism. We show that an IFN-γ driven, HIF-1α dependent signaling pathway, previously implicated in host defense, redistributes macrophage lipids into LDs. Furthermore, we show that *M*. *tuberculosis* is able to acquire host lipids in the absence of LDs, but not in the presence of IFN-γ induced LDs. This result uncouples macrophage LD formation from bacterial acquisition of host lipids. In addition, we show that IFN-γ driven LD formation supports the production of host protective eicosanoids including PGE_2_ and LXB_4_. Finally, we demonstrate that HIF-1α and its target gene *Hig2* are required for the majority of LD formation in the lungs of mice infected with *M*. *tuberculosis*, thus demonstrating that immune activation provides the primary stimulus for LD formation *in vivo*. Taken together our data demonstrate that macrophage LD formation is a host-driven component of the adaptive immune response to *M*. *tuberculosis*, and suggest that macrophage LDs are not an important source of nutrients for *M*. *tuberculosis*.

## Author summary

*Mycobacterium tuberculosis*, the causative agent of the disease tuberculosis, causes more deaths annually than any other single bacterial pathogen. *M*. *tuberculosis* primarily lives in macrophages, immune cells which specialize in phagocytosing and killing pathogens. In order to survive this inhospitable environment, *M*. *tuberculosis* must be both resistant to the bactericidal activities of macrophages and able to exploit macrophages as a replicative niche. Intracellular bacterial pathogens acquire all of their nutrients from the host cell, and *M*. *tuberculosis* relies on lipids as a key carbon source during infection. It has been proposed that lipid droplets, organelles that store neutral lipids and are observed in macrophages during *M*. *tuberculosis* infection, are an accessible nutrient source for *M*. *tuberculosis*. Furthermore, it is thought that *M*. *tuberculosis* provokes macrophages into making LDs as a pathogenic strategy. Here, we present evidence that LD formation is actually a programmed macrophage immune response, and part of the broader program of IFN-γ activation of macrophages. Furthermore, we present data uncoupling bacterial acquisition of host lipids from the presence of LDs in macrophages. Thus, we find that in addition to supporting immune functions, LDs are also unlikely to be an important nutrient source for *M*. *tuberculosis* in macrophages.

## Introduction

*Mycobacterium tuberculosis* remains a global scourge, causing more than one million deaths annually [1]. Tuberculosis is a disease characterized by long periods of latency, with viable *M*. *tuberculosis* remaining dormant in the lungs of infected patients for years to decades [2]. One of the clinical hallmarks of infection is the formation of granuloma structures in the lungs of infected patients [3,4]. Within these granulomas, where *M*. *tuberculosis* is able to persist, there are often “foamy” macrophages, which contain a large accumulation of lipid droplets (LDs) [3]. In addition to the presence of foamy macrophages, the cores of granulomas are often characterized by caseous necrosis, providing a lipid-rich environment for *M*. *tuberculosis* [5]. Not surprisingly then, *M*. *tuberculosis* devotes a large portion of its genome to lipid metabolism [6], and is capable of utilizing a variety of lipids and cholesterol as carbon sources both *in vitro* and *in vivo* [7]. Furthermore, pathways required for utilization of lipids or cholesterol as sole carbon sources are essential for *M*. *tuberculosis* growth and virulence in *in vivo* mouse models of infection [8–10].

Because *M*. *tuberculosis* requires lipids as a carbon source during infection, and infects cells which contain large numbers of LDs *in vivo*, it has been hypothesized that macrophage LDs serve as a carbon source for *M*. *tuberculosis* [3,11]. This hypothesis is supported by electron microscopy showing close apposition of LDs and phagosomes containing *M*. *tuberculosis* or *Mycobacterium avium* [3,12]. Furthermore, under conditions of hypoxia, or when exogenous oleate is added to the media, infected macrophages accumulate LDs, which correlates with acquisition of host lipids by the bacteria [3,10,13]. It has been proposed that *in vitro* macrophage LD formation during infection is dependent upon the *M*. *tuberculosis* ESX-1 secretion system, and a model has emerged wherein *M*. *tuberculosis* actively provokes and benefits from the formation of macrophage LDs, using them as a carbon source [11,14]. However, there is no direct genetic evidence indicating that the formation of host LDs is necessary for the acquisition of host lipids by *M*. *tuberculosis*. Furthermore, there is no clear consensus on signaling events in macrophages required for LD formation during *M*. *tuberculosis* infection. Numerous pathways have been proposed though, including PPAR-γ, NOTCH/MUSASHI/JMJD, and GPR109A/cAMP/PKA [11,15,16].

Although the current model for *M*. *tuberculosis* interaction with macrophage LDs during infection ascribes a passive role to LDs, LDs are not simply inert storage depots for lipids and cholesterol. They are dynamic organelles, and recent work has indicated that they play important roles in immune responses, and may be a key interface for host/pathogen interactions [17]. While they are beneficial to some pathogens, serving as an assembly site for Hepatitis C virus, and a nutrient source for *Chlamydia spp*, they have also been reported to have important immune functions, including antigen cross-presentation, viperin mediated antiviral defense, and production of pro-inflammatory eicosanoids [18]. This raises the possibility that in the context of *M*. *tuberculosis* infection, LDs play an important role in the host immune response. Indeed, it was shown that infection of macrophages with the *M*. *tuberculosis* vaccine strain Bacille Calmette-Guérin (BCG) leads to both an accumulation of LDs containing cyclooxygenase-2 (COX-2) on their surface, and enhanced production of PGE_2_, suggesting that LDs may serve as platforms for eicosanoid production during mycobacterial infection [19,20].

Control of *M*. *tuberculosis* infection requires the cytokine IFN-γ, which is produced primarily by CD4+ T cells during the adaptive response to infection [21,22]. Humans lacking components of the IFN-γ signaling pathway are extremely susceptible to mycobacterial infection [23]. While a variety of IFN-γ dependent functions which restrict *M*. *tuberculosis* growth have been proposed [24–30], the mechanisms by which IFN-γ transforms macrophages into an inhospitable environment for mycobacteria remain poorly understood. In particular, the effects of IFN-γ on macrophage metabolism during *M*. *tuberculosis* infection have only recently begun to be explored [31], and no studies have examined whether IFN-γ activation of macrophages impacts the formation and/or function of LDs during *M*. *tuberculosis* infection.

Here we demonstrate that the formation of LDs during *M*. *tuberculosis* infection *in vitro* and *in vivo* is a programmed host response that is coordinated by the cytokine IFN-γ, and that these LDs promote the production of host protective eicosanoids. We identify HIF-1α as the transcription factor through which IFN-γ dependent LD formation occurs. Interestingly, we find that HIF-1α regulates the distribution of intracellular lipids into LDs independent of total intracellular lipid levels. Furthermore, we identify *Hig2* as a HIF-1α transcriptional target that is a major regulator of LD formation in *M*. *tuberculosis* infected macrophages. Importantly, we show that this IFN-γ, HIF-1α, and *Hig2* mediated pathway for LD formation in macrophages during *M*. *tuberculosis* infection is operative *in vivo*. In addition, we find that while *M*. *tuberculosis* can readily accumulate host lipids and form bacterial lipid inclusions in resting macrophages which do not contain host LDs, *M*. *tuberculosis* has limited access to host lipids following IFN-γ activation of macrophages. Taken together, these findings suggest that macrophage LD formation during *M*. *tuberculosis* infection is a programmed host immune response, and that it is unlikely that LDs are the primary lipid source utilized by *M*. *tuberculosis* during infection.

## Results

### Macrophage LD formation during *M*. *tuberculosis* infection requires IFN-γ

**Fig 1:**
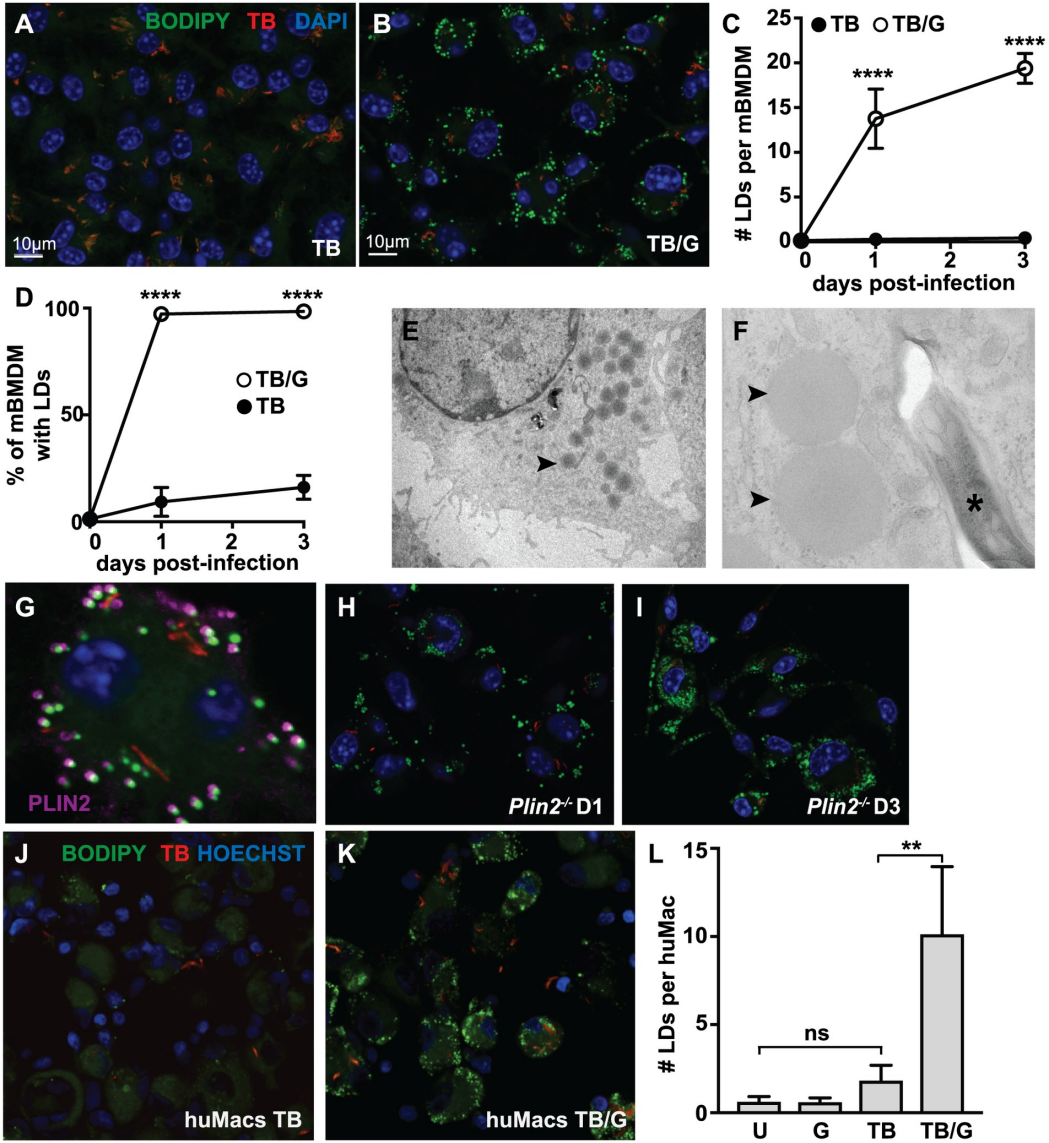
Macrophage LD formation during *M*. *tuberculosis* infection requires IFN-γ. (A) Unstimulated and (B) IFN-γ activated BMDM were infected with fluorescent *M*. *tuberculosis* 635-Turbo at MOI = 5 and imaged by confocal microscopy 3 days post-infection. Nuclei were visualized with DAPI staining and neutral lipids were visualized with BODIPY 493/503 staining. (C,D) Images from 1 and 3 days post-infection were quantified using CellProfiler for (C) the average number of LDs per BMDM and (D) the percentage of BMDM containing LDs. Each data point is quantified from ~500 BMDM. (E,F) TEM was performed on IFN-γ activated BMDMs infected with *M*. *tuberculosis* 3 days post-infection. LDs (arrowheads) and *M*. *tuberculosis* (asterisk) are indicated. (E) is at 890x magnification and (F) is at 9300x magnification. (G) IFN-γ activated BMDM were infected with fluorescent *M*. *tuberculosis* 635-Turbo. Immunofluorescence microscopy was performed 1 day post-infection, and localization of PLIN2 to BODIPY-stained structures was observed. (H,I) IFN-γ activated *Plin2*^*-/-*^ BMDM were infected with *M*. *tuberculosis* and LD formation was evaluated using BODIPY 493/503 staining 1 day post-infection (H) and 3 days post-infection (I). (J) Unstimulated and (K) IFN-γ activated primary human monocyte derived macrophages were infected with *M*. *tuberculosis* 635-Turbo at MOI = 3 and imaged by confocal microscopy 1 day post-infection. Nuclei were visualized by Hoechst 33342 and neutral lipids with BODIPY 493/503. (L) The average number of LDs per cell was quantified using CellProfiler in uninfected [U], IFN-γ activated [G], *M*. *tuberculosis* infected [TB], or IFN-γ activated and *M*. *tuberculosis* infected [TB/G] human macrophages at 1 day post-infection. Each data point is quantified from ~200 cells. All figures are representative of a minimum of three independent experiments with the exception of electron microscopy which was performed once in triplicate and human macrophages infections which were performed in duplicate. Error bars are standard deviation, **p<0.01, ****p<0.0001 by unpaired t-test.

To characterize the regulation and role of LD formation during *M*. *tuberculosis* infection, we first assessed LD formation following infection of primary murine bone marrow derived macrophages (BMDM) with the virulent Erdman strain of *M*. *tuberculosis*. Resting and IFN-γ activated BMDM were infected with fluorescent *M*. *tuberculosis* expressing 635-Turbo and LDs were identified with the neutral lipid dye BODIPY 493/503 using confocal microscopy at 0, 1, and 3 days after infection. Very few LDs accumulated in resting BMDMs infected with *M*. *tuberculosis* (Fig 1A). Quantification of this phenotype indicated that although a small fraction of BMDM had observable LDs (Fig 1D), there was an average of <1 LD per BMDM across the duration of the experiment (Fig 1C). In contrast, BMDM pre-treated with IFN-γ robustly produced LDs upon infection with *M*. *tuberculosis* (Fig 1B), with nearly 100% of BMDM containing LDs by 1 day post infection (Fig 1D), and averaging >10 LDs per macrophage (Fig 1C). Importantly, IFN-γ treatment alone did not induce LD formation (S1A Fig), indicating that macrophage LD formation is a synergistic process requiring both innate stimulation from *M*. *tuberculosis* infection and IFN-γ produced by the adaptive immune response. The TLR2 agonist Pam3CSK4 was also able to synergize with IFN-γ to induce LD formation (S1B and S1C Fig).

In IFN-γ activated, *M*. *tuberculosis* infected BMDM, transmission electron microscopy (TEM) revealed characteristic featureless spherical structures lacking a conventional membrane, consistent with bona fide LDs (Fig 1E and 1F) [32]. Perilipin proteins regulate storage of lipids in LDs by coating the surface of LDs and preventing lipolysis of the TAGs contained within the LD core. Each of the five mammalian perilipin isoforms plays a role in LD formation in a tissue and cell type specific manner [33]. RNA-seq data (SRP075696) indicated that perilipin 2 (*Plin2*) is the dominant perilipin isoform expressed in macrophages (S1F Fig). PLIN2 localized to BODIPY stained compartments in IFN-γ activated, *M*. *tuberculosis* infected BMDM (Fig 1G), further confirming the identity of the BODIPY staining structures as LDs. Interestingly, deletion of *Plin2* did not inhibit LD formation, as BMDM derived from *Plin2*^-/-^ mice did not have a defect in LD accumulation during *M*. *tuberculosis* infection with IFN-γ activation at either 1 or 3 days post-infection (Fig 1H and 1I). Next, LD formation in primary human monocyte derived macrophages was assessed. These cells had a small number of LDs at baseline (Fig 1L and S1D Fig) that was not significantly increased by treatment with IFN-γ or by *M*. *tuberculosis* infection (Fig 1J, 1L and S1E Fig). However, similar to the phenotype observed in murine BMDM, the combination of IFN-γ treatment and *M*. *tuberculosis* infection resulted in a robust accumulation of LDs in primary human macrophages (Fig 1K and 1L).

### *M*. *tuberculosis* acquisition of host lipids does not correlate with LD formation in infected macrophages

**Fig 2:**
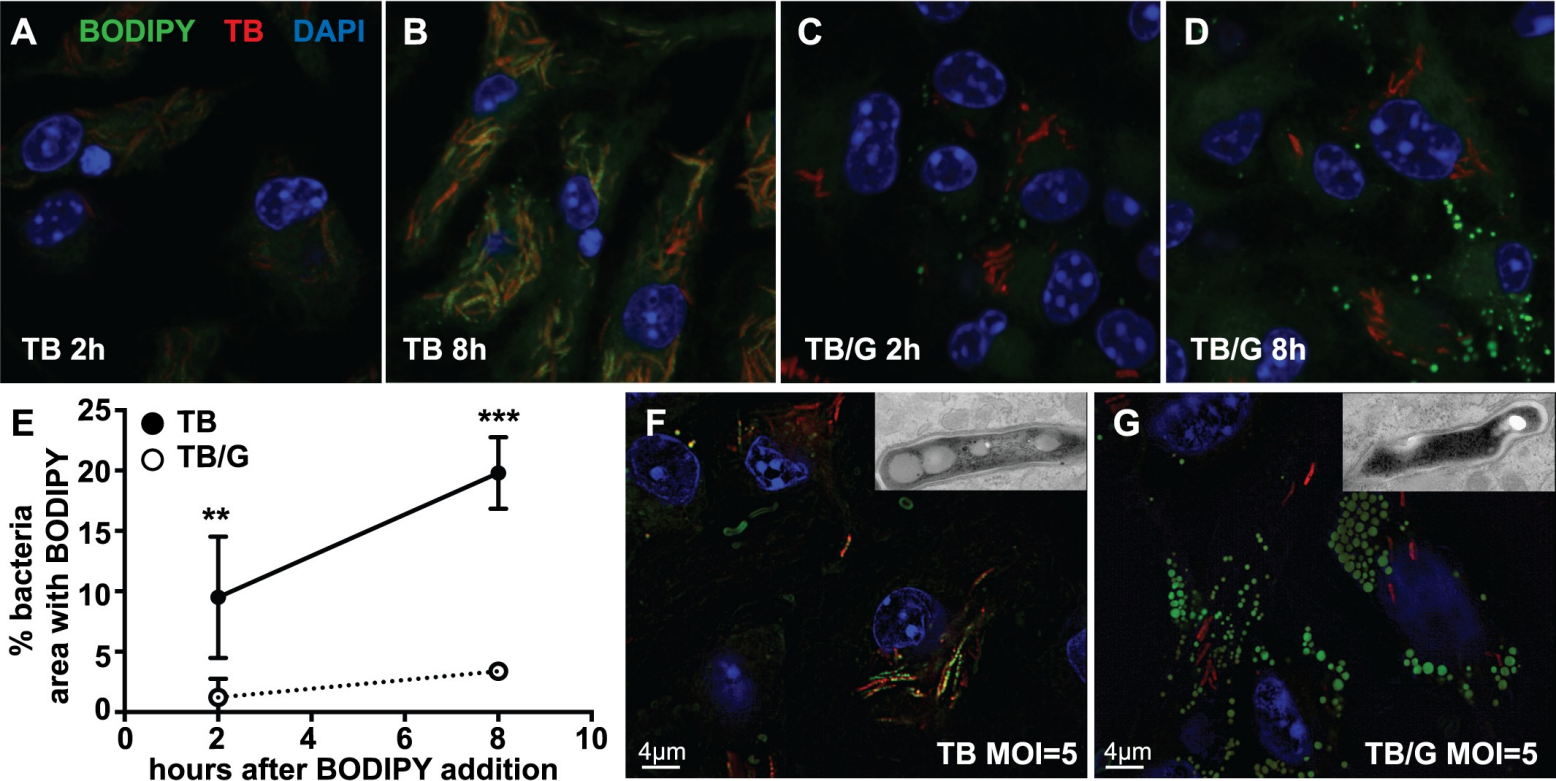
*M*. *tuberculosis* acquisition of host lipids does not correlate with LD formation in infected macrophages. (A-D) Fluorescently labeled fatty acid BODIPY FL C16 was added to media of *M*. *tuberculosis* 635-Turbo infected BMDM 3 days post-infection. Bacterial lipid accumulation was analyzed by confocal microscopy at 2 hours and 8 hours after BODIPY FL C16 addition in the absence of IFN-γ activation (A,B), and with IFN-γ activation (C,D). (E) Bacterial accumulation of BODIPY FL C16 during *M*. *tuberculosis* infection of BMDM without IFN-γ activation [TB] and with IFN-γ activation [TB/G] was quantified using CellProfiler from images as in (A-D). Each data point is quantified from bacteria contained within ~100 infected BMDM. (F) BMDM were infected with *M*. *tuberculosis* 635-Turbo in the absence of IFN-γ. 3 days post-infection, BMDM were fixed, BODIPY 493/503 stained, and analyzed by SIM. Punctate BODIPY 493/503 staining co-localizing with *M*. *tuberculosis* was observed. TEM of infected BMDM at the same time point shows that these BODIPY 493/503 puncta are bacterial lipid inclusions (inset). (G) IFN-γ activated BMDM were infected and analyzed as in (F). SIM and TEM imaging indicates that in IFN-γ activated BMDM, BODIPY 493/503 signal does not localize with *M*. *tuberculosis* and bacterial lipid inclusions are not present (inset). All figures are representative of three experiments with the exception of TEM, which was performed once in triplicate. Error bars are standard deviation, **p<0.01, ***p<0.001 by unpaired t-test.

To determine whether the ability of *M*. *tuberculosis* to acquire host lipids correlates with macrophage LD formation, BMDM infected with *M*. *tuberculosis* were pulsed with a fluorescently labeled fatty acid, BODIPY FL C16, by addition to the media. Within 2 hours, BODIPY FL C16 accumulated inside *M*. *tuberculosis* in resting BMDM, and this accumulation increased over an 8 hour timecourse (Fig 2A and 2B). Notably, this bacterial lipid accumulation occurred in the absence of macrophage LDs (Fig 2A and 2B). In contrast, there was minimal accumulation of BODIPY FL C16 inside *M*. *tuberculosis* in IFN-γ activated BMDM where host LDs are observed (Fig 2C and 2D). BODIPY FL C16 accumulation inside of *M*. *tuberculosis* was quantified by the percentage of bacterial area identified by 635T expression that stained with BODIPY FL C16 (Fig 2E). To determine whether the observed BODIPY signal localizing to *M*. *tuberculosis* results from cell wall staining or accumulation of lipids inside the bacteria, both Structured Illumination Microscopy (SIM) and TEM were performed. SIM demonstrated that the bacterially associated BODIPY signal in resting BMDM infected with *M*. *tuberculosis* is punctate and co-localizes with bacteria (Fig 2F). This staining is not apparent in IFN-γ activated BMDM infected with *M*. *tuberculosis*, where strong macrophage LD staining is observed (Fig 2G). In addition, TEM images identified structures inside the bacteria characteristic of bacterial lipid inclusions which corroborated the SIM imaging results (Fig 2F and 2G insets) [34]. Taken together, these results indicate that in the absence of macrophage LDs, *M*. *tuberculosis* is able to readily accumulate lipid inclusions, and that under conditions where IFN-γ has induced macrophage LD formation, the ability of *M*. *tuberculosis* to accumulate lipid inclusions is impaired.

### LD formation is associated with an increase in intracellular triglycerides and cholesterol esters, and requires the fatty acid translocase CD36

**Fig 3:**
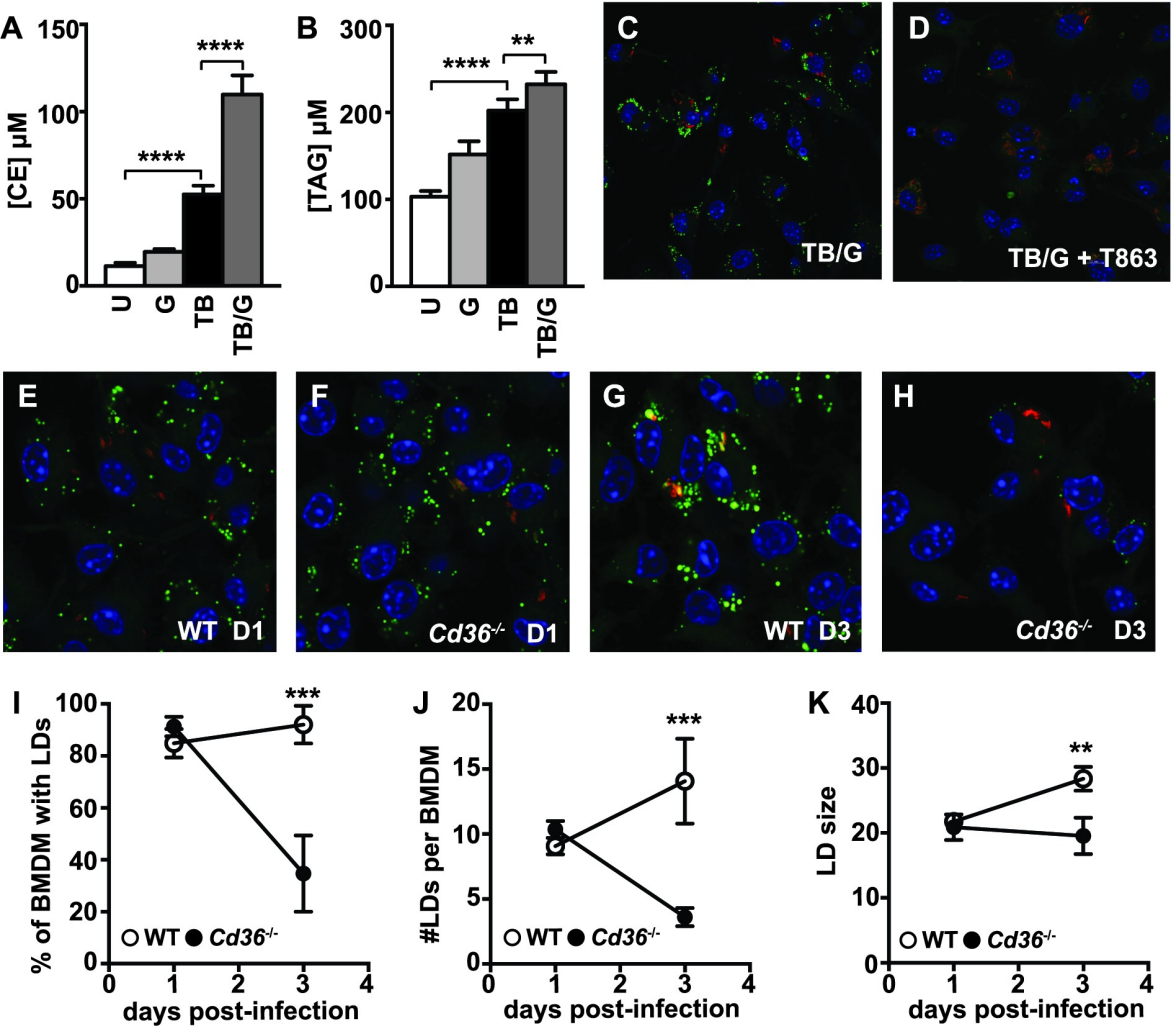
LD formation is associated with an increase in intracellular triacylglycerols and cholesterol esters, and requires the fatty acid translocase CD36. Lipidomic quantification of (A) total cellular cholesterol ester [CE] and (B) total triacylglycerol [TAG] concentrations in wildtype BMDM 1 day post-infection was performed by Metabolon, Inc using mass spectrometric analysis of quintuplicate samples. BMDM were either uninfected [U], IFN-γ activated [G], *M*. *tuberculosis* infected [TB], or IFN-γ activated and *M*. *tuberculosis* infected [TB/G]. (C,D) IFN-γ activated BMDM were infected with *M*. *tuberculosis* and the DGAT1 inhibitor T863 was added after the 4 hour phagocytosis period (D). Microscopy was performed 1 day post-infection. (E-H) IFN-γ activated wildtype and *Cd36*^-/-^ BMDM were infected with *M*. *tuberculosis* 635-Turbo. BODIPY 493/503 staining and confocal microscopy was performed 1 day post-infection (E,F) and 3 days post-infection (G,H). CellProfiler was used to quantify (I) the percentage of BMDM with LDs, (J) the number of LDs per BMDM, and (K) LD size. Each data point is quantified from ~500 BMDM. (C-K) Figures are representative of three independent experiments. (A,B) Lipidomic profiling was performed once in quintuplicate. Error bars are standard deviation, **p<0.01, ***p<0.001, ****p<0.0001 by unpaired t-test.

The robust accumulation of macrophage LDs observed during *M*. *tuberculosis* infection with IFN-γ activation suggests that there are major changes in lipid metabolism. To test whether LD formation under these conditions is associated with changes in the abundance of cellular lipids, mass spectrometry was performed to measure the abundance of a wide range of lipids (S1 Table). Levels of most phospholipids and sphingolipids were unchanged in BMDM during infection with *M*. *tuberculosis*, in BMDM following IFN-γ activation, or in BMDM activated with IFN-γ and infected with *M*. *tuberculosis* (S1 Table). However, there were significant changes in levels of TAGs and cholesterol esters, the primary components of LDs, which were elevated during infection with *M*. *tuberculosis* and further increased by IFN-γ activation (Fig 3A and 3B). Of note however, is the observation that total TAG levels are only slightly higher in BMDM activated with IFN-γ and infected with *M*. *tuberculosis* compared to resting BMDM infected with *M*. *tuberculosis* (Fig 3B). This result suggests that elevation of TAG levels alone is insufficient to induce LD formation in macrophages.

Next, the source of the increased lipids observed during *M*. *tuberculosis* infection was examined. LDs bud from the ER membrane, and their formation and maintenance results from a balance of TAG and cholesterol import, synthesis, export and lipolysis [35]. RNA-seq data demonstrated that the primary fatty acid synthase, *Fasn*, was dramatically downregulated in BMDM during *M*. *tuberculosis* infection with IFN-γ activation (S2A Fig), suggesting that *de novo* fatty acid (FA) synthesis is not a major driver of LD accumulation in macrophages during *M*. *tuberculosis* infection. Independent of the source of FAs, formation of TAGs requires the activity of the acyl-CoA:diacylglycerol acyltransferase (DGAT) enzymes DGAT1 and DGAT2, which catalyze the final step in TAG synthesis [36,37]. Treatment of *M*. *tuberculosis* infected, IFN-γ activated BMDM with the DGAT1 inhibitor T863 [38] prevented LD formation during the first 24 hours after infection (Fig 3C and 3D) and this defect in LD formation continued through 3 days after infection (S2B and S2C Fig). Similarly, DGAT1 inhibition in primary human macrophages prevented LD formation during the first 24 hours after infection (S2D and S2E Fig). These results indicate that TAG synthesis but not FA synthesis contributes to LD formation during *M*. *tuberculosis* infection of IFN-γ activated macrophages.

CD36, a scavenger receptor also known as fatty acid translocase, is a receptor for lipoprotein particles such as LDL and VLDL and long chain fatty acids [39]. CD36 has been shown to contribute to lipid import under M2 differentiating conditions and is important for uptake of surfactant by human macrophages infected with *M*. *tuberculosis* [40,41]. Furthermore, *Cd36*^-/-^ mice were shown to be resistant to BCG infection [42]. Interestingly, RNA-seq analysis revealed that *Cd36* expression is increased by IFN-γ treatment of *M*. *tuberculosis* infected BMDM (S2F Fig), suggesting that this import pathway might play a role in macrophage LD formation during *M*. *tuberculosis* infection. While no defect in LD formation in *Cd36*^-/-^ BMDM infected with *M*. *tuberculosis* and activated with IFN-γ was observed 1 day post-infection (Fig 3E and 3F), *Cd36*^-/-^ BMDM had a striking defect in LDs 3 days post infection (Fig 3G and 3H). This defect included a dramatic decrease in the percent of BMDM containing LDs (Fig 3I), the average number of LDs per BMDM (Fig 3J), and the average size of LDs (Fig 3K). Taken together, these data demonstrate that LD formation requires TAG synthesis at all time points, and that the maintenance of LDs, but not their initial formation, requires import of lipids via CD36.

### HIF-1α is required for LD formation during *M*. *tuberculosis* infection

**Fig 4:**
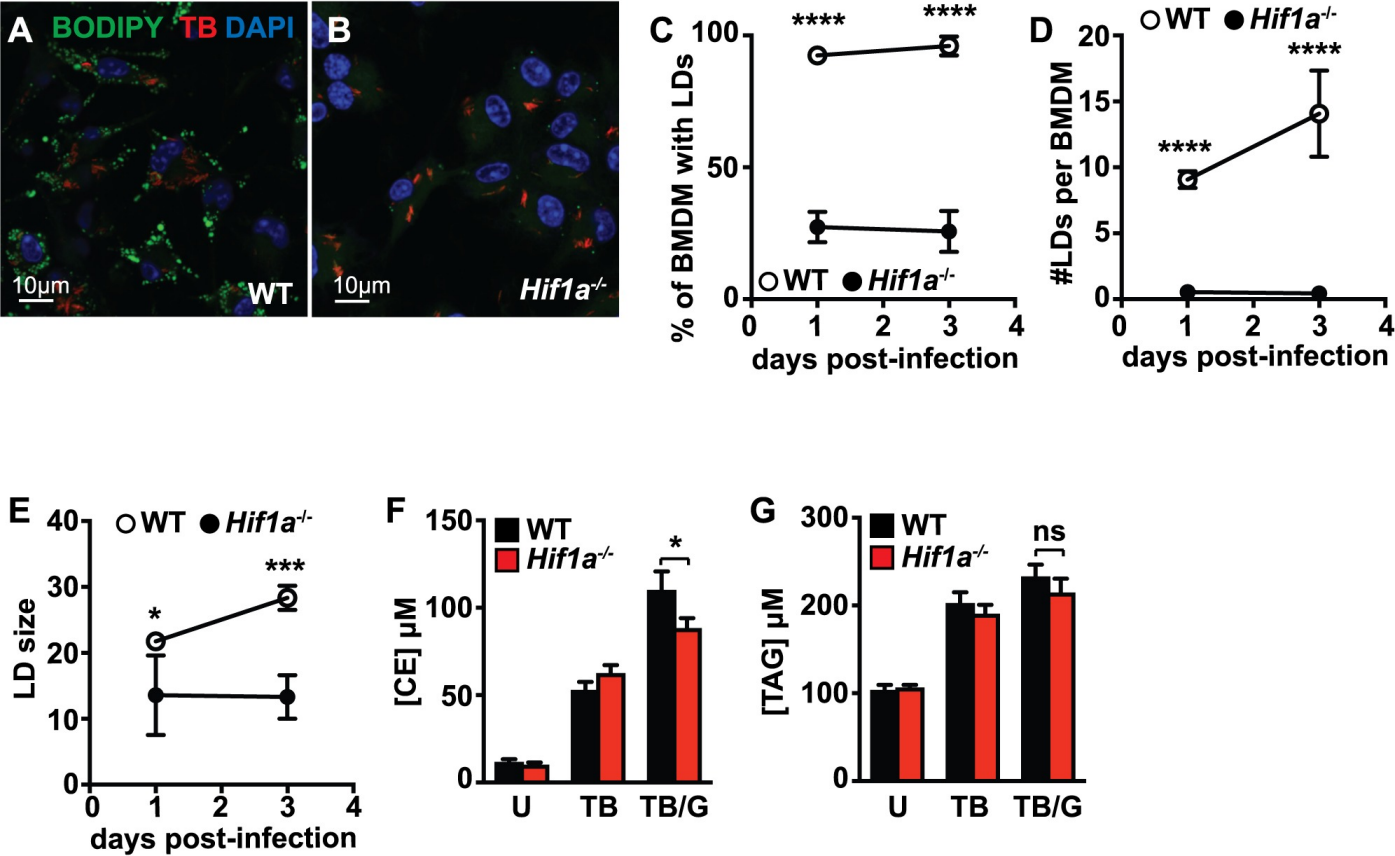
HIF-1α is required for LD formation during *M*. *tuberculosis* infection. (A) Wildtype and (B) *Hif1a*^-/-^ BMDMs were infected with *M*. *tuberculosis* 635-Turbo following IFN-γ activation. 1 day post-infection, BMDM were stained with BODIPY 493/503 and confocal microscopy was performed. CellProfiler was used to quantify (C) the percentage of BMDM with LDs, (D) the number of LDs per BMDM, and (E) LD size 1 and 3 days post-infection. Each data point is quantified from ~500 BMDM. (F,G) Lipidomic quantification of (F) total cellular cholesterol ester [CE] and (G) total triacylglycerol [TAG] concentrations in wildtype and *Hif1a*^-/-^ BMDM was performed by Metabolon, Inc using mass spectrometric analysis of quintuplicate samples. BMDM were infected with *M*. *tuberculosis* and cell lysates were prepared 1 day post-infection. BMDM were either uninfected [U], *M*. *tuberculosis* infected [TB], or IFN-γ activated and *M*. *tuberculosis* infected [TB/G]. (A-E) Figures are representative of a minimum of three experiments. (F,G) Lipidomic profiling was performed once in quintuplicate. Error bars are standard deviation, *p<0.05, ***p<0.001, ****p<0.0001 by unpaired t-test.

The transcription factor HIF-1α, the master regulator of the hypoxia response, has been shown to mediate LD formation during hypoxia [43,44]. In macrophages it has been demonstrated that HIF-1α can be induced by immune stimuli such as LPS treatment or infection with a variety of bacterial pathogens [45–48]. We recently demonstrated that HIF-1α is important for control of *M*. *tuberculosis* infection *in vitro* and *in vivo*, and that the activation of HIF-1α during *M*. *tuberculosis* infection is almost entirely IFN-γ dependent [31]. These observations suggest that HIF-1α might mediate IFN-γ dependent LD formation during *M*. *tuberculosis* infection. Indeed, LD formation in *Hif1a*^*-/-*^ BMDM was severely compromised (Fig 4A and 4B). In the absence of HIF-1α, a much lower percentage of macrophages had LDs (Fig 4C), there was a dramatic decrease in the number of LDs per macrophage (Fig 4D), and the few LDs that did accumulate were smaller (Fig 4E). However, lipidomic analysis in *Hif1a*^*-/-*^ BMDM revealed only a slight defect in cholesterol ester accumulation (Fig 4F), and no statistically significant defect in TAG accumulation (Fig 4G). These data indicate that accumulation of lipids is not sufficient for LD formation, and suggests that HIF-1α target genes may mediate localization of neutral lipids into cytosolic LDs. Previously, we found that iNOS expression and NO production are required for HIF-1α protein stabilization and transcriptional activation of target genes in IFN-γ activated macrophages infected with *M*. *tuberculosis* [49]. In agreement with this data, BMDM lacking iNOS (*Nos2*^-/-^) also have a significant defect in LD accumulation following IFN-γ activation and *M*. *tuberculosis* infection (S3A and S3B Fig). Similarly, *Nos2*^*-/-*^ BMDM treated with Pam3CSK4 and IFN-γ have a defect in LD accumulation (S3C and S3D Fig).

### HIF-1α target gene *Hig2* is required for LD maintenance during *M*. *tuberculosis* infection

**Fig 5:**
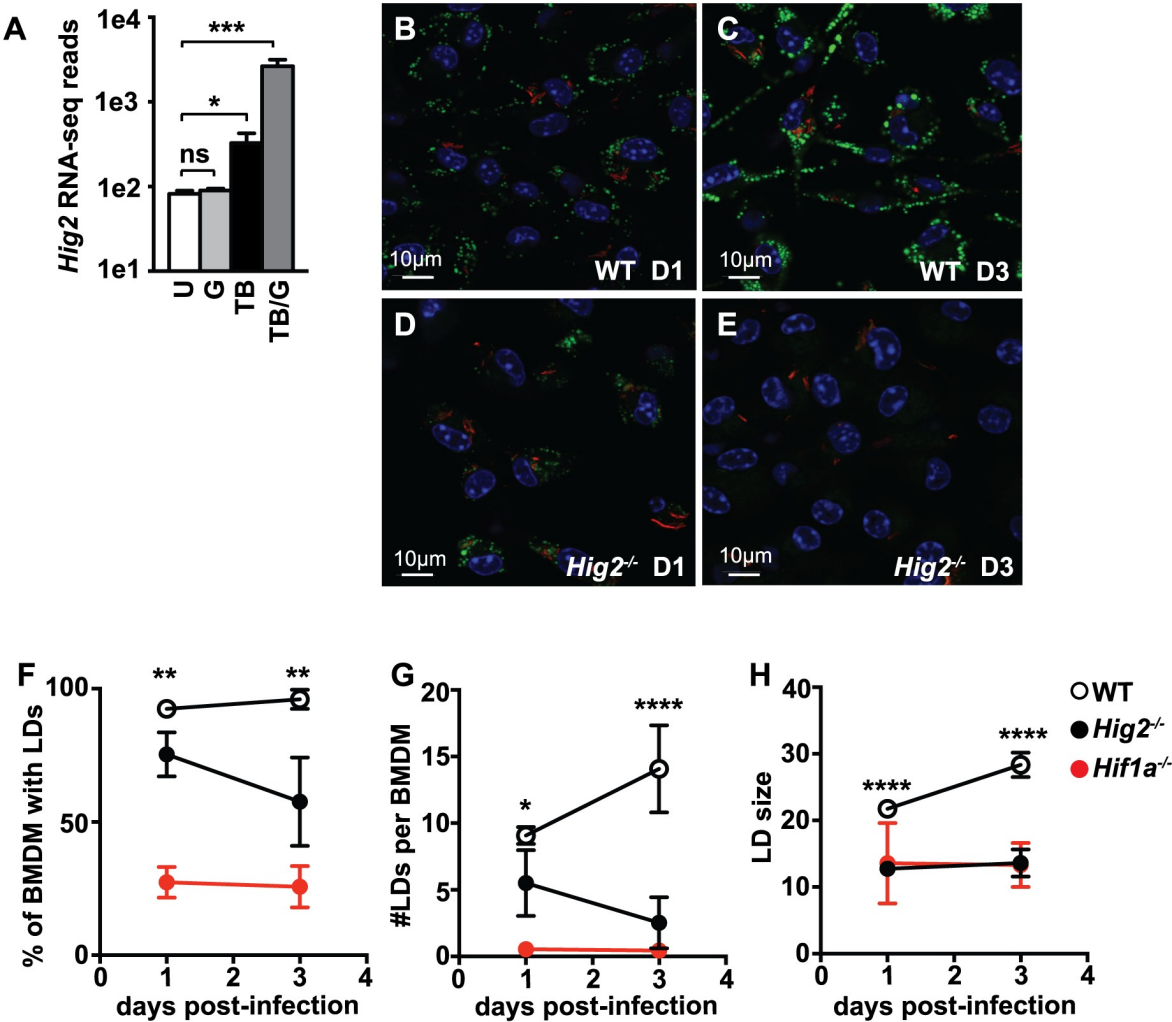
HIF-1α target gene *Hig2* is required for LD maintenance during *M*. *tuberculosis* infection. (A) RNA-seq data for transcript levels of *Hig2* in wildtype BMDM either unstimulated [U], IFN-γ activated [G], *M*. *tuberculosis* infected [TB], or IFN-γ activated and *M*. *tuberculosis* infected [TB/G]. Samples were collected 1 day post-infection in 3 independent experiments. (B-E) IFN-γ activated wildtype and *Hig2*^-/-^ BMDM were infected with *M*. *tuberculosis* 635-Turbo. BODIPY 493/503 staining and confocal microscopy was performed 1 day post-infection (B,D) and 3 days post-infection (C,E). CellProfiler was used to quantify (F) the percentage of BMDM with LDs, (G) the number of LDs per BMDM, and (H) LD size. Each data point is quantified from ~500 BMDM. Figures are representative of three experiments, error bars are standard deviation, *p<0.05, **p<0.01, ***p < .001, ****p<0.0001 by unpaired t-test.

HIF-1α is responsible for ~50% of the changes in gene expression in IFN-γ activated, *M*. *tuberculosis* infected BMDM [31]. These HIF-1α regulated genes were examined to identify candidate genes that specifically mediate LD formation during *M*. *tuberculosis* infection. Canonical LD associated genes were equivalently expressed in wildtype and HIF-1α deficient BMDM, including *Pnpla2*, *Plin2*, and *Plin3* (S4A Fig). However, expression of Hypoxia Inducible Gene-2 (*Hig2*) was strongly and synergistically induced by the combination of *M*. *tuberculosis* infection and IFN-γ activation (Fig 5A), and this transcriptional upregulation was almost entirely HIF-1α dependent (S4A Fig). Previously, HIG2 was shown to localize to LDs in a model of non-alcoholic fatty liver disease, and mutating *Hig2* in this system caused LD abnormalities [50]. Furthermore, *Hig2* was identified as a HIF-1α target gene [43]. Thus, we hypothesized that the HIF-1α dependent upregulation of *Hig2* in IFN-γ activated macrophages infected with *M*. *tuberculosis* contributes to LD accumulation.

To test whether *Hig2* contributes to LD accumulation during *M*. *tuberculosis* infection, *Hig2* was first deleted in BMDM using CRISPR/Cas9. *Cas9* transgenic bone marrow was transduced with lentivirus encoding an sgRNA that targets exon 2 of the *Hig2* gene, and these transduced bone marrow cells were differentiated into BMDM. *Hig2* mRNA levels were substantially decreased in BMDM which had been targeted with *Hig2* sgRNA relative to control sgRNA (S4B Fig). Furthermore, BMDM targeted with *Hig2* sgRNA had a defect in LD formation during *M*. *tuberculosis* infection with IFN-γ activation (S4C and S4D Fig). Next, the same sgRNA sequence was used to generate *Hig2*^-/-^ mice using CRISPR/Cas9. The resulting founder mice were screened for *Hig2* mutations by PCR followed by sequencing. A founder with a 19-bp frameshift deletion in exon 2 was used to establish a pure homozygous *Hig2*^*-/-*^ line (S4E Fig). LD accumulation was then assessed in wildtype and *Hig2*^-/-^ BMDM during *M*. *tuberculosis* infection with IFN-γ activation. At 1 day post-infection, there was a modest defect in LD accumulation in *Hig2*^-/-^ BMDM relative to wildtype BMDM (Fig 5B and 5D), and this defect became much more pronounced at 3 days post-infection (Fig 5C and 5E). Quantification of this phenotype demonstrated that a lower percentage of *Hig2*^-/-^ BMDM had LDs (Fig 5F). Furthermore, by 3 days post-infection, *Hig2*^-/-^ BMDM had a nearly 90% defect in the average number of LDs per cell compared to wildtype BMDM, nearly as complete a defect as that observed in *Hif1a*^*-/-*^ BMDM (Fig 5G). Finally, at all timepoints analyzed, the average size of the LDs in *Hig2*^-/-^ BMDM was smaller than those observed in wildtype BMDM, recapitulating the LD size phenotype observed in *Hif1a*^*-/-*^ BMDM (Fig 5H).

### LDs support host immunity in *M*. *tuberculosis* infected macrophages

**Fig 6:**
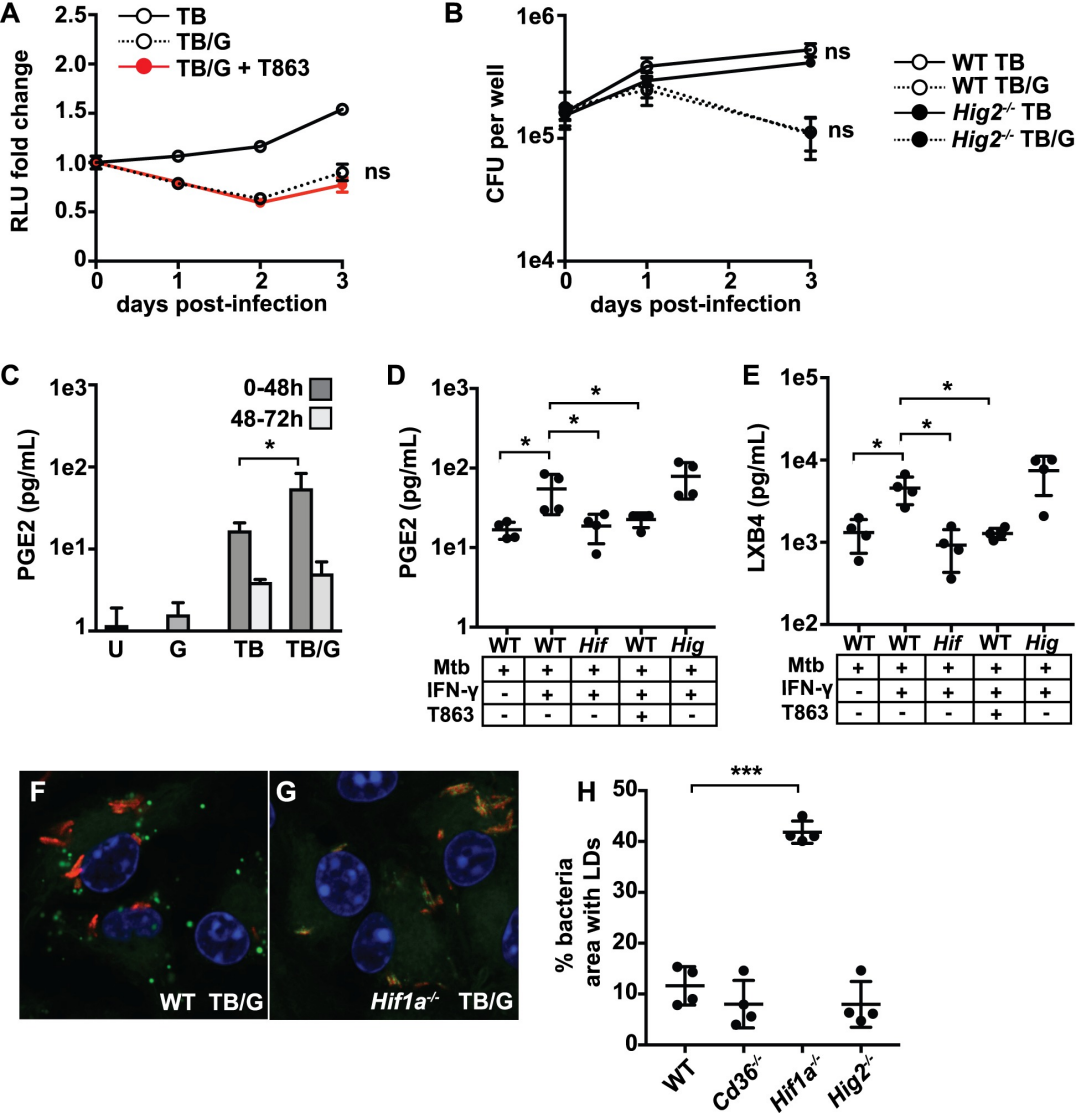
LDs support host immunity in *M*. *tuberculosis* infected and IFN-γ activated macrophages. (A) Resting and IFN-γ activated BMDM were infected with TB-Lux *M*. *tuberculosis* at MOI = 5, and T863 was added after the 4 hour phagocytosis. Lux readings were taken at day 0 (after the 4 hour phagocytosis), and days 1, 2, and 3 post-infection. Data is normalized to the day 0 read. (B) Wildtype and *Hig2*^-/-^ BMDM were infected as in (A) and bacterial numbers were enumerated by plating for CFU at the indicated time points. (C) LC-MS/MS measurement of PGE_2_ in supernatants from wildtype BMDM either unstimulated [U], IFN-γ activated [G], *M*. *tuberculosis* infected [TB], or IFN-γ activated and *M*. *tuberculosis* infected [TB/G]. Supernatant samples were taken in quadruplicate at both 48 hours post-infection and again at 72 hours post-infection from the same cells. (D,E) LC-MS/MS measurement of PGE_2_ (D) and LXB_4_ (E) from supernatants of BMDM infected with *M*. *tuberculosis*, 48 hours post-infection. Samples are from wildtype [WT], *Hif1a*^-/-^ [*Hif*] and *Hig2*^-/-^ [*Hig*] BMDM, with IFN-γ and T863 treatment as indicated. (F,G) Wildtype and *Hif1a*^-/-^ BMDM were activated with IFN-γ and infected with *M*. *tuberculosis* 635-Turbo and stained with BODIPY 493/503 at 3 days post-infection. (H) Quantification of bacterial area colocalizing with lipid staining at 3 days post-infection in wildtype, *Cd36*^*-/-*^, *Hif1a*^*-/-*^, and *Hig2*^*-/-*^ BMDM activated with IFN-γ and infected with *M*. *tuberculosis*. Figures are representative of a minimum of three experiments, except for LC-MS/MS eicosanoid profiling which was performed once in quadruplicate. Error bars are standard deviation. *p<0.05, ***p<0.001 by unpaired t-test.

Next, *M*. *tuberculosis* growth in macrophages was assessed during pharmacological or genetic inhibition of LD formation. Treatment of IFN-γ activated, *M*. *tuberculosis* infected BMDM with the DGAT1 inhibitor T863 to block LD formation did not change *M*. *tuberculosis* replication as assayed by a luminescent reporter strain of *M*. *tuberculosis* (Fig 6A) [31]. Similarly, no differences in CFU were observed in *Hig2*^-/-^ BMDM compared to wildtype BMDM infected with *M*. *tuberculosis*, either in the presence or absence of IFN-γ (Fig 6B). These data demonstrate that macrophage LDs are neither essential for *M*. *tuberculosis* growth nor required for cell intrinsic control of infection in this system.

Eicosanoid production has a significant impact on the outcome of *M*. *tuberculosis* infection *in vivo*, with PGE_2_ and lipoxins amongst the specific eicosanoids with a demonstrated role [30,51–53]. Our previous work showed that HIF-1α is required for the majority of PGE_2_ production during *M*. *tuberculosis* infection of IFN-γ activated BMDM [31]. *Hif1a*^*-/-*^ BMDM have a modest defect in transcript levels of *Cox2* during infection, which could explain a defect in production of PGE_2_. However, we hypothesized that *Hif1a*^*-/-*^ BMDM are defective in eicosanoid production additionally due to their lack of LD accumulation. To test this hypothesis, LC-MS/MS based eicosanomic profiling was performed on supernatants from BMDM under a variety of *M*. *tuberculosis* infection conditions including genetic and pharmacological inhibition of LD formation to assess total eicosanoid production (S2 Table). In this *in vitro* system production of eicosanoids occurred during the first two days after infection, dropping dramatically after 48hr (Fig 6C and S2 Table). For a wide array of eicosanoids, production was induced by *M*. *tuberculosis* infection alone, but substantially elevated by the addition of IFN-γ (S2 Table). For PGE_2_ in particular, IFN-γ alone did not induce PGE_2_ production, but it significantly enhanced PGE_2_ production during *M*. *tuberculosis* infection (Fig 6C). Thus, PGE_2_ production by BMDM correlates with LD formation during *M*. *tuberculosis* infection. *Hif1a*^*-/-*^ BMDM were found to be deficient in the production of a broad array of arachidonic acid derived eicosanoids including HETEs, prostaglandins, and lipoxins (S5A–S5E Fig and S2 Table), including PGE_2_ and LXB_4_ (Fig 6D and 6E). In accordance with the model that LDs are involved in macrophage production of eicosanoids during infection, inhibition of LD formation with T863 phenocopied the HIF-1α deficient BMDM for production of prostaglandins and LXB_4_ (Fig 6D, 6E and S5D, S5E Fig). Interestingly, T863 did not impair production of HETEs (S5A–S5C Fig), suggesting that prostaglandin and lipoxin production is specifically enhanced by LD formation. Taken together, the data suggest that LDs are an important site of eicosanoid biosynthesis that enhances the production of prostaglandins and lipoxins during *M*. *tuberculosis* infection. Somewhat surprisingly, there was no defect in eicosanoid production in *Hig2*^-/-^ BMDM (Fig 6D, 6E and S5A–S5E Fig). This may be because the decreased accumulation of LDs in *Hig2*^-/-^ BMDM is only partial at early timepoints when the bulk of eicosanoid production occurs, and at late timepoints where the *Hig2*^-/-^ BMDM have a dramatic defect in LDs there is only minimal eicosanoid production (Fig 6C).

### *M*. *tuberculosis* is unable to acquire host lipids in IFN-γ activated macrophages that express HIF-1α

We next sought to determine if the ability of *M*. *tuberculosis* to accumulate lipids is restored in IFN-γ activated BMDM in which LD formation is inhibited. Because inhibition of DGAT1 leads to LD defects as a secondary consequence of disrupted TAG synthesis, bacterial acquisition of lipids was examined in *Hif1a*^*-/-*^ BMDM, which have equivalent TAG levels as wildtype BMDM (Fig 4G). Wildtype and *Hif1a*^*-/-*^ BMDM were infected with *M*. *tuberculosis* for 3 days and lipid accumulation by bacteria was determined by BODIPY 493/503 labeling. As expected, at 3 days after infection numerous mammalian LDs were observed in the cytosol of *M*. *tuberculosis* infected and IFN-γ activated wildtype BMDM, and no BODIPY staining was evident on bacteria (Fig 6F and 6H). However, in *Hif1a*^*-/-*^ BMDM, *M*. *tuberculosis* regained the ability to accumulate lipids as evidenced by robust BODIPY 493/503 staining (Fig 6G and 6H). Bacteria infecting *Cd36*^*-/-*^ and *Hig2*^*-/-*^ BMDM, which have only partial defects in LD formation, phenocopied infection of wildtype BMDM (Fig 6H).

### LDs are not an important source of nutrients for *M*. *tuberculosis* growth *in vivo*

**Fig 7:**
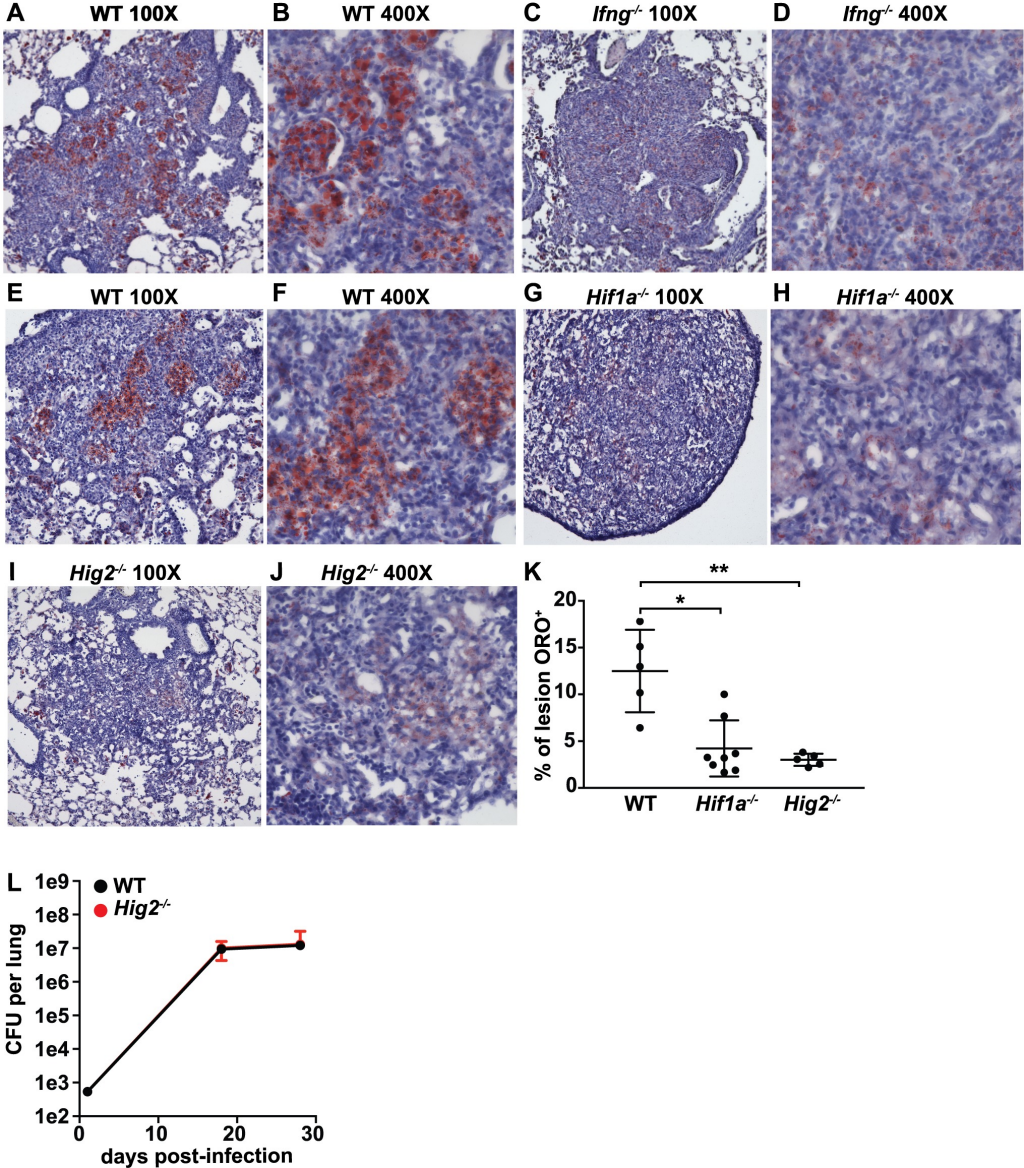
IFN-γ signaling is required for LD formation during *M*. *tuberculosis* infection *in vivo*. **(A-J)** Mice were infected with ~200 CFU of *M*. *tuberculosis* via the aerosol route and lungs were collected for histological analysis of LD formation by Oil Red O (ORO) staining. (A-D) ORO stained lung sections from wildtype and *Ifng*^-/-^ mice 21 days post-infection. Sections were counter-stained with hematoxylin. (E-J) ORO stained lung sections from wildtype (E,F), *LysMcre*^*+/+*^*; Hif1a*^*fl/fl*^ (G,H), and *Hig2*^-/-^ mice (I,J) at 28 days post-infection. (K) ORO signal as a percentage of lesion area was quantified in sections from wildtype, *LysMcre*^*+/+*^*; Hif1a*^*fl/fl*^, and *Hig2*^-/-^ mice. (L) Bacterial burden in the lungs of wildtype and *Hig2*^-/-^ mice was enumerated by plating for CFU at 1, 18 and 28 days post-infection. Images and figures are representative of at least 3 independent experiments. Error bars are standard deviation, *p<0.05, **p<0.01 by unpaired t-test.

Next, we tested whether IFN-γ driven, HIF-1α dependent expression of *Hig2* is required for LD formation in lung lesions during *in vivo M*. *tuberculosis* infection. Wildtype, *Ifng*^*-/-*^, *LysMcre*^*+/+*^*; Hif1a*^*fl/fl*^, and *Hig2*^*-/-*^ mice were infected with the virulent *M*. *tuberculosis* strain Erdman via the aerosol route and LDs were identified by Oil Red O (ORO) neutral lipid staining on lung sections. Wildtype mice had substantial ORO staining in lung lesions at 21 and 28 days post-infection (Fig 7A, 7B, 7E and 7F). At 21 days post-infection, lesions from *Ifng*^*-/-*^ mice had fewer LDs compared to wildtype mice (Fig 7A–7D), suggesting that the IFN-γ dependent pathway for LD formation we identified in BMDM is also operative *in vivo*. Similarly, at 28 days post infection, *LysMcre*^*+/+*^*; Hif1a*^*fl/fl*^ and *Hig2*^*-/-*^ mice had dramatically reduced ORO staining in lung lesions compared to wildtype mice (Fig 7E–7K). Both *LysMcre*^*+/+*^*; Hif1a*^*fl/fl*^ and *Hig2*^*-/-*^ mice had faint (but not entirely absent) ORO staining (Fig 7H and 7J). Higher magnification images revealed that the LDs observed in *Hig2*^*-/-*^ lesions were fewer in number and substantially smaller in size than those seen in wildtype lesions (S6A and S6B Fig). Interestingly, *Hig2*^*-/-*^ mice appear to have no defect in the presence of LDs in lung epithelial cells outside of lesions (S6C and S6D Fig).

We next sought to determine whether the lack of lipid droplets in *Hig2*^*-/-*^ mice would impact *in vivo* growth of *M*. *tuberculosis* as a result of altered nutrient availability. Because mutants that are unable to subsist on lipids as a sole carbon course have been shown to be attenuated during early stages of infection [8,54], we measured bacterial burden in the lungs at 18 and 28 days post infection. We observed no differences in CFU between wildtype and *Hig2*^*-/-*^ mice at these timepoints (Fig 7L). Taken together, these results indicate that the IFN-γ/HIF-1α/*Hig2* pathway for LD production during *M*. *tuberculosis* infection is operative *in vivo* as well as *in vitro*, and is responsible for lesion specific LD formation. Furthermore, these results strongly suggest that LDs are not a source of nutrients required for *M*. *tuberculosis* growth *in vivo*.

## Discussion

LDs have recently received significant attention in the context of infection and immunity. Although there is evidence to suggest that LDs play a role in promoting immune responses, LDs have primarily been investigated as a source of nutrients for multiple intracellular pathogens, including *M*. *tuberculosis*, *Chlamydia spp*, *and Toxoplasma gondii* [18,55]. A model has emerged for the role of LDs in the context of *M*. *tuberculosis* infection of macrophages wherein virulent strains of *M*. *tuberculosis* induce LD formation in macrophages and then utilize these LDs as a carbon source [11,14,17]. Surprisingly, in this study we find that in both primary murine macrophages and primary human macrophages, *M*. *tuberculosis* infection with a virulent strain does not robustly induce LD formation. Instead, we find that during *M*. *tuberculosis* infection, macrophage LD formation is part of an adaptive immune response activated by IFN-γ. Furthermore, we find that in the absence of macrophage LDs, *M*. *tuberculosis* is able to accumulate lipid inclusions and that the addition of IFN-γ and accompanying production of macrophage LDs appears to limit bacterial lipid accumulation. These data suggest that macrophage LDs are not the major source from which *M*. *tuberculosis* acquires host lipids. In this study we also explore the mechanisms by which macrophages regulate LD formation during infection, and elucidate the signaling pathway that leads to LD formation downstream of IFN-γ activation. We find that the transcription factor HIF-1α and its target gene *Hig2* are required for LD formation, a finding that correlates LD formation with activation of a host protective mechanism. Additionally, we show that inhibiting LD formation using the DGAT1 inhibitor T863 does not impact bacterial replication in resting macrophages or in IFN-γ activated macrophages, but does lead to a decrease in production of arachidonic acid derived metabolites, including PGE_2_. Finally, we show that IFN-γ/HIF-1α/*Hig2* signaling is operative *in vivo*. Although *Hig2* is required for LD formation in lung lesions in mice infected with *M*. *tuberculosis*, we observed no difference in the bacterial burden in the lungs of *Hig2*^*-/-*^ mice compared to wildtype controls. This result suggests that *in vivo*, LDs are not a nutrient source that is required for *M*. *tuberculosis* growth.

Several lines of evidence indicate that *M*. *tuberculosis* utilizes host lipids during intracellular infection. First, bacterial genes required for utilization of non-sugar carbon sources are essential for growth in *in vitro* macrophage infection models and in *in vivo* murine infection models [8,56]. Second, *M*. *tuberculosis* is capable of using lipids as a sole carbon source in axenic culture, and accumulates lipids derived from host macrophages when LD formation is stimulated by either hypoxia or by addition of exogenous fatty acids [10,13]. Third, LD laden foamy macrophages are a characteristic feature of human granulomas. Indeed, *in vitro* conditions that simulate the granuloma by using human primary PBMCs that include T cells and macrophage-like cells result in robust LD accumulation in macrophages [3,57,58]. These multiple lines of indirect evidence have provided support for a model in which *M*. *tuberculosis* acquires lipids directly from host LDs. The findings presented here challenge this model, and indicate that *M*. *tuberculosis* may be able to acquire lipids from non-LD sources.

Although EM studies have shown an occasional apposition of *M*. *tuberculosis* containing phagosomes with LDs in *in vitro* granulomas [3], other studies demonstrate a segregation of *M*. *tuberculosis* from neutral lipid staining compartments [58] which is similar to what we have observed. Indeed, we find that IFN-γ treatment results in macrophage LDs that do not co-localize with *M*. *tuberculosis*, suggesting that these LDs do not serve as an accessible source of nutrients under these conditions. Furthermore, we find that in IFN-γ activated wildtype macrophages which accumulate LDs, *M*. *tuberculosis* no longer accumulates bacterial lipid inclusions. Importantly, we used a concentration of IFN-γ that results in bacteriostatic rather than bactericidal activity, suggesting that the majority of bacteria under these conditions are viable but non-replicating. This stands in stark contrast to infection of resting macrophages, where *M*. *tuberculosis* replicates, macrophage LDs do not form, and *M*. *tuberculosis* readily acquires lipids and forms large numbers of bacterial lipid inclusions. Thus, the formation of macrophage LDs is inversely correlated with both bacterial LD acquisition and bacterial growth. Interestingly, in *Hif1a*^*-/-*^ macrophages activated with IFN-γ, where there is defective killing of *M*. *tuberculosis* [31], we observe a restoration of bacterial lipid inclusions. Future work will determine whether the lack of bacterial lipid inclusions in wildtype macrophages results from a HIF-1α dependent anti-microbial mechanism, or simply reflects changes in bacterial metabolism that result from IFN-γ mediated immune pressure.

Because *M*. *tuberculosis* is likely capable of obtaining host lipids from numerous abundant lipid rich sources *in vivo*, including the phagosomal membrane during intracellular growth and necrotic tissue during extracellular growth, we propose that there is no requirement for the utilization of macrophage LDs for *M*. *tuberculosis* to obtain host lipids. LDs form under numerous conditions, and their function and trafficking is dictated by the composition of the proteins embedded in their phospholipid monolayer. It is therefore possible that LDs with different characteristics could form in *M*. *tuberculosis* infection in response to stimuli other than IFN-γ. For example, exposure of macrophages to hypoxia, phagocytosis of apoptotic cells and/or necrotic debris, or stimulation with extracellular free fatty acids or VLDL could all lead to LD formation *in vivo*. So it is possible that a subset of LDs may be accessible to *M*. *tuberculosis* containing phagosomes *in vivo*. However, our *in vivo* studies suggest that during *M*. *tuberculosis* infection LDs primarily form in response to IFN-γ driven HIF-1α activation, and subsequent *Hig2* transcription in macrophages in lung lesions.

HIF-1α has a major role in metabolic reprogramming in response to a wide variety of stimuli, and has been shown to mediate LD formation during hypoxia via transcriptional activation of several gene products, including fatty acid importers, adipophilin, and *Hig2* [43,44,59]. In hepatocytes *Hig2* has been proposed to inhibit lipolysis [50], whereas in adipocytes *Hig2* is a PPAR-γ dependent gene that associates with LDs but does not regulate lipolysis. We found that in macrophages infected with *M*. *tuberculosis* and stimulated with IFN-γ, HIF-1α regulates the storage of lipids in LDs but not the import of fatty acids. However, macrophages lacking HIF-1α have a nearly complete defect in LD accumulation. Because the defect in LD accumulation in *Hig2*^*-/-*^ macrophages is partial, there is at least one more HIF-1α dependent regulator of IFN-γ dependent LD formation that remains to be discovered. Although we find that CD36 is required for the maintenance of LDs in macrophages, *Cd36* is not a transcriptional target of HIF-1α under these conditions.

We and others have shown that HIF-1α is an important mediator of host defense in macrophages, and HIF-1α deficient mice are extremely susceptible to infection with *M*. *tuberculosis*. We previously found that HIF-1α activates expression of numerous host protective factors, including iNOS, IL-1, and COX-2 leading to PGE_2_ production [31]. Here we show that HIF-1α mediated LD formation impacts the production of numerous arachidonic acid derived metabolites, including PGE_2_ and LXB_4_. These data suggest that LDs could play a greater role in host defense against *M*. *tuberculosis* infection than has previously been appreciated. Although HIF-1α deficient mice are susceptible to *M*. *tuberculosis* infection, the pleiotropic phenotypes observed in HIF-1α deficient mice during infection makes it impossible to determine whether the lack of LDs contributes to this failure of host defense.

In this study, we did not observe a failure to control *M*. *tuberculosis* infection in *Hig2*^*-/-*^ mice at 18 days or 28 days post-infection. However, this data may not fully reflect the role of LDs in eicosanoid production. First, *Ptgs2* deficient animals that are unable to make host protective PGE_2_ only show susceptibility to *M*. *tuberculosis* over timeframes that are significantly longer than 28 days [53]. Thus longer experiments may reveal a phenotype in *Hig2*^*-/-*^ mice. Second, we found that ablation of LDs results in changes in levels of numerous eicosanoids, the functions of which have not been studied in *M*. *tuberculosis* infection. It is possible that eicosanoids produced on LDs have antagonistic functions in the context of *M*. *tuberculosis* infection. Finally, although we were able to ablate the vast majority of LD formation in macrophages in infected murine lungs by mutation of *Hig2*, we were unable to completely eliminate LD formation. Identification of additional HIF-1α dependent target genes and complete ablation of LD formation *in vivo* will facilitate a complete analysis of the role of LDs in *M*. *tuberculosis* infection. Finally, it was somewhat surprising that abrogation of PGE_2_ production by the DGAT inhibitor T863 did not result in a defect in cell intrinsic control in isolated macrophages in our hands, a result that contrasts with previous reports [51,53]. It is possible that the conditions used in our experiments do not reveal the cell protective effects of PGE_2_, or that that the pleiotropic effects of DGAT inhibition abrogate these effects.

Taken together, the findings reported here indicate that rather than being a bacterially driven process utilized by *M*. *tuberculosis* for successful nutrient acquisition and replication, LD formation is a programmed IFN-γ dependent macrophage response to infection that enhances eicosanoid production. We find that innate immune stimulation by *M*. *tuberculosis* synergizes with IFN-γ activation to induce LD formation in macrophages, and that TLR2 stimulation with Pam3CSK4 similarly synergizes with IFN-γ to induce LD formation. Interestingly, the *M*. *tuberculosis* cell wall lipid trehalose dimycolate (TDM) is a TLR ligand that has been associated with LD formation in previous studies [3,5,60]. Thus, it is possible that TDM is the innate immune stimulus that IFN-γ synergizes with to induce LDs during *M*. *tuberculosis* infection. Furthermore, we elucidate the signaling pathway through which these macrophage LDs are regulated downstream of receptor stimulation. We identify an IFN-γ/HIF-1α/*Hig2* pathway operative both *in vitro* in primary macrophages, and in an *in vivo* aerosol model of *M*. *tuberculosis* infection. Additionally, we find that these IFN-γ induced LDs enhance the macrophage immune response by serving as an important site for production of a broad range of immunomodulatory eicosanoids. Interestingly, these results may also have relevance in human disease: granuloma-like structures from patients with latent tuberculosis infection have greater macrophage accumulation of LDs than those obtained from healthy controls, suggesting that antigen experienced T cells may produce a factor that promotes LD formation *in vivo* [58]. In this granuloma model the T cells produce IFN-γ, suggesting that the pathway we describe here for LD formation during *M*. *tuberculosis* infection may also be operational during human disease. In support of this model, we find that in primary human monocyte derived macrophages, IFN-γ treatment synergizes with *M*. *tuberculosis* infection to induce LD formation.

## Materials and methods

### Ethics statement

All procedures involving the use of mice were approved by the University of California, Berkeley IACUC, the Animal Care and Use Committee (protocol number R353-1113B). All protocols conform to federal regulations, the National Research Council’s Guide for the Care and Use of Laboratory Animals and the Public Health Service’s (PHS’s) Policy on Humane Care and Use of Laboratory Animals.

### Reagents

Recombinant mouse IFN-γ (485-MI/CF), recombinant human IFN-γ (285-IF), recombinant human GM-CSF (215-GM), and Pam3CSK4 (4633) were obtained from R&D systems. 1α,25-Dihydroxyvitamin D_3_ (BML-DM200) was obtained from Enzo Life Sciences. T863 (SML0539) and Histopaque 1077 (10771) were obtained from Sigma-Aldrich. BODIPY 493/503 (D3922), BODIPY FL C16 (D3821), HCS LipidTOX Red (H34476), Hoechst 33342 (H3570), and ProLong Diamond Antifade Mountant (P36965) were obtained from ThermoFisher. Primary polyclonal PLIN2 antibody (AP5118C) was obtained from ABGENT.

### Mice

Wildtype C57BL/6 mice were obtained from Jackson Laboratory, and then bred in house. All knockout mice are on the C57BL/6 background. B6.129-*Hif1a*^*tm3Rsjo*^*/J* (HIF1α^flox^) mice were obtained from the Jackson Laboratory and were crossed with B6.129P2-*Lyz2*^*tm1(cre)Ifo*^*/J* (LysMcre) to generate *Hif1a*^flox/flox^,LysMcre^+/+^ mice that had *Hif1a* deletion targeted to the myeloid lineage. B6.129S7-*Ifng*^*tm1Ts*^/J (Ifng^*-/-*^) and B6.129P2-*Nos2*^*tm1Lau*^*/J* (*Nos2* ^-/-^) mice were obtained from the Jackson Laboratory and were bred in house. *Hig2*^*-/-*^ mice were created as described in the text in collaboration with the Cancer Research Laboratory at UC Berkeley. *Cd36*^-/-^ mice were a kind gift from Maria Febbraio and bred in house.

### Cell culture

Murine BMDM were derived by flushing the bone marrow from femurs and tibias and culturing these cells in DMEM with 10% FBS, 2 mM L-glutamine and 10% supernatant from 3T3-M-CSF cells for 6 days with media addition on day 3. After differentiation, BMDM were cultured in DMEM supplemented with 10% FBS, 2mM GlutaMAX, and 10% supernatant from 3T3-M-CSF cells (BMDM media). Peripheral blood mononuclear cells (PBMCs) were isolated from buffy coats by centrifugation over Histopaque-1077. Total PBMCs were then plated on Poly D Lysine coated 96 well plates with 5x10^5^ cells per well and differentiated for 14 days in RPMI supplemented with 10% FBS, 1mM Sodium Pyruvate, 1mM NEAA, 2mM GlutaMAX, and 10 ng/mL GM-CSF (PBMC media) with a 50% media change every 2–3 days. 25 ug/mL kanamycin was added to the media during the first week of differentiation.

### Bacterial culture

The *M*. *tuberculosis* strain Erdman was used for all experiments. *M*. *tuberculosis* was grown in Middlebrook 7H9 liquid media supplemented with 10% ADS (albumin-dextrose-saline), 0.4% glycerol, and 0.05% Tween-80. The fluorescent 635-Turbo strain used for microscopy and the luminescent TB-lux strain used for measuring bacterial growth are derived from an Erdman strain and were cultured as described above.

### *In vitro* infections

BMDM were plated into 96-well or 24-well plates with 5x10^4^ and 3x10^5^ macrophages per well respectively, and were allowed to adhere and rest for 24 hours. For all experiments where IFN-γ was used, BMDM were pretreated with recombinant mouse IFN-γ overnight (at 6.25ng/ml unless otherwise indicated) and then infected in DMEM supplemented with 5% horse serum and 5% FBS at an MOI of 5. After a 4 hour phagocytosis period, infected BMDM were washed with PBS before replacing with BMDM media. For IFN-γ pretreated wells, IFN-γ was also added after phagocytosis at the same concentration. For experiments where T863 was used, it was added at 2.5uM after the end of the 4 hour phagocytosis.

For IFN-γ treatment of PBMCs, cells were pretreated overnight with recombinant human IFN-γ (5ng/mL) and vitamin D (100uM). PBMCs were infected in RPMI supplemented with 5% horse serum and 5% FBS at an MOI of 3. After the 4 hour phagocytosis period, cells were washed with PBS before replacing with PBMC media. For wells pretreated with IFN-γ, IFN-γ and vitamin D were added after phagocytosis at the same concentrations.

For enumeration of CFU, infected BMDM were washed with PBS, lysed in water with 0.5% Triton-X for ten minutes at 37C, and serial dilutions were prepared in PBS with 0.05% Tween-80 and plated onto 7H10 plates. To measure bacterial growth by luminescence, BMDM were infected with the TB-lux strain and luminescence was measured at the end of the 4 hour phagocytosis after a PBS wash and media replacement. Luminescence was subsequently read at the indicated timepoints, and growth was normalized to the initial luminescence readings for each infected well.

### Metabolomics

Complex lipid profiling by was performed by Metabolon, Inc. BMDMs were seeded in quintuplicate in 15cm dishes with 2.5x10^7^ cells per dish and infected at an MOI of 5. 24 hours post-infection, cells were harvested by scraping and lipids were extracted via Bligh-Dyer extraction protocol. Media was removed, a mixture of 0.9 ml water and 2 ml of methanol was added to the cells in conicals, and immediately placed on ice. Samples were transferred to glass test tubes, 900ul of dichloromethane and 100 ul of Metabolon internal standard mixture was added and samples were gently vortexed for 5 seconds, followed by incubation for 30 min at room temperature. A mixture of 1ml water and 900ul dichloromethane was added, samples were again gently vortexed and then centrifuged at 2000g for 10 min to create a bi-layer. The bottom chloroform layer was transferred to a new glass tube and dried under nitrogen. Samples were reconstituted in 600ul of a 1:1 mixture of dichloromethane and methanol for mass spectrometry analysis.

For eicosanoid profiling, BMDM were plated in a 24 well plate at a density of 3x10^5^ BMDM per well in a volume of 1mL, with 4 replicate wells per condition. 24 hours after plating, media was changed to either BMDM media, or BMDM media with 3.125 ng/mL IFN-γ. After overnight IFN-γ pretreatment, BMDM were infected with *M*. *tuberculosis* (Erdman) at an MOI of 5. Following the 4 hour phagocytosis period, media was changed and IFN-γ was added again at 3.125ng/mL to all IFN-γ pre-treated wells. For DGAT inhibition, T863 was added at 2.5uM after the end of the 4 hour phagocytosis period. There was no pretreatment with T863, and it was only added following infection to avoid any alterations in lipid metabolism prior to infection. 48 hours after infection, supernatants were collected for eicosanomic profiling. 400uL of supernatant was added to 800uL of ice cold 100% MeOH. After pipetting gently to mix, samples were transferred to -80C. A media change was performed at 48 hours post-infection, with IFN-γ and T863 added again at the same concentrations. A second round of supernatants was collected 72 hours post-infection, to measure eicosanoid production between 48 hours and 72 hours post-infection. Eicosanoids and PUFA were quantified via liquid chromatography- tandem mass spectrometry (LC-MS/MS) according to published protocols[61,62]. Briefly; class specific deuterated internal standards PGE_2_-d4, LTB4-d4, 15-HETE-d8, LXA_4_-d5, DHA-d5, AA-d8 (Cayman Chemical) were used to calculate work up and extraction recovery. The LC-MS/MS system consisted of an Agilent 1200 Series HPLC, Kinetex C18 minibore column (Phenomenex), and an AB Sciex QTRAP 4500 mass spectrometer. Analyses were carried out in negative ion mode, and eicosanoids and PUFA were identified and quantified by scheduled multiple reaction monitoring (MRM) using 4–6 specific transition ions for each analyte.

### Microscopy

For confocal imaging of BMDM, cells were plated on glass coverslips in 24 well plates at a density of 3x10^5^ BMDM per coverslip, and infected with *M*. *tuberculosis* Erdman-635T. 24 hours or 72 hours post-infection, coverslips were fixed in 10% formalin for 1 hour, washed with PBS, and stained with DAPI and BODIPY 493/503 each at a concentration of 1 ug/ml in PBS for 1 hour. For live labeling with a fluorescent lipid, BMDM media with 1 ug/ml BODIPY FL C16 was added to cells at indicated time points and cells were then fixed and DAPI stained as previously described. Coverslips were mounted on slides with an antifade mounting media and allowed to set overnight. Imaging was done on a Carl Zeiss LSM710 confocal microscope. Images shown were taken with a 63x objective. For quantification, larger fields were taken with a 20x objective. For confocal imaging of PBMCs, infected cells were washed with PBS, fixed in 4% paraformaldehyde for 1 hour, washed with PBS, stained with 1 ug/ml Hoechst 33342 for 10 minutes, and then stained with 1 ug/ml BODIPY 493/503 for 1 hour. Imaging was done on a Perkin Elmer Opera Phenix Automated Microscopy System. Images were taken with a 40x objective.

For immunofluorescence, BMDMs were infected on coverslips as described before, and fixed for 1 hour with 4% paraformaldehyde. Coverslips were washed with PBS and incubated in PBS blocking buffer with 3% BSA, 1.5% glycine, and .01% (w/v) saponin for 2 hours at room temperature. Coverslips were washed again with PBS and incubated with primary antibody diluted 2000-fold in a PBS solution of 0.1% BSA and 0.01% (w/v) saponin, overnight at 4C. The following day, secondary Alexafluor antibody was diluted 2000-fold, and added to coverslips for 2 hours. Coverslips were then washed and mounted on slides for confocal imaging. For SIM, BMDM were plated on Zeiss high performance coverslips (#474030-9000-000) in 6 well plates with 1.5x10^6^ cells per coverslip, and infected with *M*. *tuberculosis* Erdman-635T. 72 hours post-infection, coverslips were fixed and stained, as previously described, and mounted on slides with ProLong Diamond Antifade Mountant. Microscopy was performed on a Carl Zeiss Elyra SR.1 Super Resolution microscope. For TEM, BMDM were plated on plastic aclar coverslips in 24 well plates and infected as previously described. 3 days post-infection, coverslips were fixed in 4% PFA and 2% glutaraldehyde for 24 hours, rinsed with 0.1M cacodylate buffer, postfixed in cacodylate buffer with 1% osmium tetroxide and 1.6% potassium ferricyanide, and stained with 2% uranyl acetate. Coverslips were ethanol dehydrated in 70%, 90%, and 100% ethanol, then resin infiltrated in 50% resin 50% acetone, 75% resin 25% acetone, followed by pure resin and incubated overnight at 60C. Samples were thin-sectioned and imaged on a FEI Tecnai 12 transmission electron microscope. Microscopy was performed at The CNR Biological Imaging Facility and the Electron Microscope Lab at The University of California, Berkeley.

### *In vivo* infections

Cohorts of age and sex matched wildtype, *Hif1a*^flox/flox^ LysMcre^+/+^, *Hig2*^*-/-*^, and *Ifng*^*-/-*^ mice were infected by aerosol route with *M*. *tuberculosis* strain Erdman at a dose of ~200 CFU. All mice were on the C57BL/6 background, and were 7–12 weeks of age when infected. Aerosol infection was done using a Nebulizer and Full Body Inhalation System (Glas-Col) as previously described [31]. For CFU enumeration from lungs, the left lobe was collected, homogenized in PBS plus 0.05% Tween 80, and serial dilutions were plated on 7H10 plates. For lung histology samples, the right inferior lobe from each mouse was fixed in 10% formalin overnight at room temperature and then incubated at room temperature in a sucrose gradient of 10% sucrose for 1 hour, 20% sucrose for 1 hour, and 30% sucrose overnight at 4C. Lungs were then dried on Kimwipes and placed in base molds, covered in OCT, and frozen with dry ice and isopentane. Samples were thin-sectioned and stained with Oil Red O and hematoxylin counterstain at the Gladstone Histology and Light Microscopy Core.

## Supporting information

S1 Fig IFN-γ induces LD formation in the context of TLR2 stimulation and induces increased *Plin2* expression during *M*. *tuberculosis* infection. Wildtype BMDM were treated with (A) IFN-γ, (B) Pam3CSK4, or (C) IFN-γ and Pam3CSK4 for 24 hours, fixed, and imaged by confocal microscopy. Pam3CSK4 was used at 50ng/mL. Nuclei were stained with DAPI and neutral lipids were stained with BODIPY 493/503. (D,E) Primary human monocyte derived macrophages were (D) unstimulated or (E) IFN-γ treated for 24 hours, fixed, and imaged by confocal microscopy. Nuclei were stained with Hoechst 33342 and neutral lipids with BODIPY 493/503. (F) RNA-seq data showing transcript levels of members of the PLIN/PAT protein family in wildtype BMDM 24 hours post-infection in uninfected [U], *M*. *tuberculosis* infected [TB], or IFN-γ activated and *M*. *tuberculosis* infected [TB/G] BMDM. Figures are representative of a minimum of three experiments with the exception of human macrophage experiments which were performed in duplicate. Error bars are standard deviation. **p<0.01 by unpaired t-test.
(PDF)

S2 Fig TAG synthesis rather than *de novo* fatty acid synthesis is required for LD formation in macrophages. (A) RNA-seq data showing transcript levels of *Fasn* in wildtype BMDM 24 hours post-infection in uninfected [U], IFN-γ activated [G], *M*. *tuberculosis* infected [TB], or IFN-γ activated and *M*. *tuberculosis* infected [TB/G] BMDM. (B,C) IFN-γ activated BMDM were infected with *M*. *tuberculosis* 635-Turbo and the DGAT1 inhibitor T863 was added after the 4 hour phagocytosis period (C). BMDM were imaged 3 days post-infection by confocal microscopy. Nuclei were stained with DAPI and neutral lipids were stained with BODIPY 493/503. (D,E) IFN-γ activated primary human monocyte derived macrophages were infected with *M*. *tuberculosis* 635-Turbo and the DGAT1 inhibitor T863 was added after the 4 hour phagocytosis period (E), and were imaged 1 day post-infection by confocal microscopy. Nuclei were stained with Hoechst 33342 and neutral lipids were stained with BODIPY 493/503. (F) RNA-seq data showing transcript levels of *Cd36* in wildtype BMDM 24 hours post-infection. Figures are representative of a minimum of three independent experiments, with the exception of human macrophage experiments which were performed in duplicate. Error bars are standard deviation. **p<0.01, ***p < .001 by unpaired t-test.
(PDF)

S3 Fig *Nos2* is required for LD formation during *M*. *tuberculosis* infection. (A,B) Wildtype and *Nos2*^-/-^ BMDM were activated with IFN-γ and infected with *M*. *tuberculosis* 635-Turbo. Nuclei were stained with DAPI and neutral lipids were stained with LipidTox Green. (C,D) Wildtype and *Nos2*^*-/-*^ BMDM were treated with Pam3CSK4 and IFN-γ. Nuclei were stained with DAPI and neutral lipids were stained with LipidTox Green. Figures are representative of a minimum of three independent experiments.
(PDF)

S4 Fig *Hig2* knockout with CRISPR/Cas9 in macrophages and mice. (A) RNA-seq data showing expression levels of LD associated genes in wildtype and *Hif1a*^-/-^ BMDM uninfected [U] and IFN-γ activated and *M*. *tuberculosis* infected [TB/G]. Timepoint is 24 hours post-infection. (B) Cas9 transgenic BMDM stably expressing single guides targeting *Hig2* or *Cd11b* were infected with *M*. *tuberculosis-*GFP with and without IFN-γ activation. RNA was isolated and qPCR data is shown for expression of *Hig2* normalized to actin (*Actb*). (C,D) Cas9 transgenic BMDM stably expressing single guides targeting *Hig2* (C) or *Cd11b* (D) were activated with IFN-γ and infected with *M*. *tuberculosis-*GFP. 1 day post-infection, BMDM were fixed, nuclei were stained with DAPI, LDs were stained with LipidTox Red, and widefield microscopy was performed. (E) A *Hig2*^-/-^ mouse was generated using CRISPR/Cas9 and one sgRNA targeted to the *Hig2* exon that induced a 19 nucleotide deletion and frameshift mutation. Sequence and chromatogram of the wildtype *Hig2* and the *Hig2* deletion allele are shown. Error bars are standard deviation, **p<0.01, ***p<0.001 by unpaired t-test.
(PDF)

S5 Fig Macrophage eicosanoid production is partially dependent on LD formation. (A-E) LC-MS/MS based eicosanoid profiling was performed on BMDM supernatants 48 hours post-infection with *M*. *tuberculosis* in wildtype [WT], *Hif1a*^*-/-*^ [*Hif*], and *Hig2*^*-/-*^ [*Hig*] BMDM. IFN-γ and DGAT1 inhibitor T863 treatment was added as indicated. Data is shown for the following eicosanoids: (A) 5-HETE, (B) 15-HETE, (C) 12-HETE, (D) PGD2, and (E) PGF2A. Eicosanoid profiling was performed once in quadruplicate. Error bars are standard deviation, *p<0.05, **p<0.01 by unpaired t-test.
(PDF)

S6 Fig LD defects in *Hig2*^-/-^ mice are lesion specific. Mice were infected with ~200 CFU of *M*. *tuberculosis* Erdman via the aerosol route and lungs were collected for histological analysis of LD formation by Oil Red O (ORO) staining. Sections were counterstained with hematoxylin. (A) ORO accumulation in wildtype lung lesions as in Fig 7E and 7F, but with higher resolution and digital zoom. (B) ORO accumulation in *Hig2*^*-/-*^ lung lesions as in Fig 7I and 7J. Higher resolution images show that in *Hig2*^*-/-*^ lesions, the LDs that are present are smaller. (C,D) ORO staining LDs were observed in the epithelium in non-lesion areas in wildtype (C) and *Hig2*^*-/-*^ (D) lungs, with no apparent difference between genotypes.
(PDF)

S1 Table Cellular lipid profile. Lipidomic quantification of cellular lipids from wildtype and *Hif1a*^*-/-*^ BMDM that were untreated (U), IFN-γ activated (G), *M*. *tuberculosis* infected (TB), or IFN-γ activated and *M*. *tuberculosis* infected (TB/G). Samples were prepared 24 hours post-infection. Values listed are the average uM concentration from 5 biological replicates, with standard deviation listed in parentheses. Lipids profiled are cholesteryl ester (CE), triacylglycerol (TAG), diacylglycerol (DAG), free fatty acid (FFA), phosphatidylcholine (PC), phosphatidylethanolamine (PE), phosphatidylinositol (PI), lysophosphatidylcholine (LPC), lysophosphatidylethanolamine (LPE), sphingomyelin (SM), ceramide (CER), hexosylceramide (HCER), lactosylceramide (LCER), dihydroceramide (DCER).
(DOCX)

S2 Table Macrophage eicosanoid production. Lipidomic quantification of eicosanoids from untreated wildtype BMDM (WT U), IFN-γ activated wildtype BMDM (WT G), *M*. *tuberculosis* infected wildtype BMDM (WT TB), IFN-γ activated and *M*. *tuberculosis* infected wildtype BMDM (WT TB/G), IFN-γ activated and *M*. *tuberculosis* infected *Hif1a*^*-/-*^ BMDM (*Hif* TB/G), IFN-γ activated and *M*. *tuberculosis* infected wildtype BMDM treated with T863 (WT T863), and IFN-γ activated and *M*. *tuberculosis* infected *Hig2*^*-/-*^ BMDM (*Hig* TB/G). Values listed are the average concentrations in pg/ml from 4 biological replicates, with standard deviations listed in parentheses. For each eicosanoid listed, the value in the top row represents the concentration at 48 hours post-infection and value in the bottom row represents the concentration at 72 hours post-infection (following a media change at 48 hours post-infection). Eicosanoids listed: arachidonic acid (AA); 5-, 12-, and 15-hydroxyeicosatetraenoic acid (5-,12-, 15-HETE); prostaglandin E2 (PGE2); prostaglandin D2 (PGD2); prostaglandin F2alpha (PGF2a); lipoxin A4 (LXA4); lipoxin B4 (LXB4); thromboxane B2 (TXB2); docosahexaenoic acid (DHA); 4-, 7-, 14-, and 17-hydroxy docosahexanoic acid (4-, 7-, 14-, 17-HDHA); eicosapentaenoic acid (EPA); 12-, 15-, and 18-hydroxyeicosapentaenoic acid (12-, 15-, 18-HEPE); 13-hydroxyoctadecadienoic acid (13-HODE). *N.D. = not detected.
(DOCX)

## References

[1] Floyd K. Global Tuberculosis Report 2016. Geneva: World Health Organization; 2016 12 pp. 1–214.

[2] Getahun H, Matteelli A, Chaisson RE, Raviglione M. Latent Mycobacterium tuberculosis infection. The New England journal of medicine. 2015;372: 2127–2135. doi: 10.1056/NEJMra1405427 26017823 10.1056/NEJMra1405427

[3] Peyron P, Vaubourgeix J, Poquet Y, Levillain F, Botanch C, Bardou F, et al Foamy macrophages from tuberculous patients' granulomas constitute a nutrient-rich reservoir for M. tuberculosis persistence. PLOS Pathogens. 2008;4: e1000204 doi: 10.1371/journal.ppat.1000204 19002241 10.1371/journal.ppat.1000204 PMC2575403

[4] Russell DG, Cardona P-J, Kim M-J, Allain S, Altare F. Foamy macrophages and the progression of the human tuberculosis granuloma. Nat Immunol. 2009;10: 943–948. doi: 10.1038/ni.1781 19692995 10.1038/ni.1781 PMC2759071

[5] Kim M-J, Wainwright HC, Locketz M, Bekker L-G, Walther GB, Dittrich C, et al Caseation of human tuberculosis granulomas correlates with elevated host lipid metabolism. EMBO Mol Med. 2010;2: 258–274. doi: 10.1002/emmm.201000079 20597103 10.1002/emmm.201000079 PMC2913288

[6] Cole ST, Brosch R, Parkhill J, Garnier T, Churcher C, Harris D, et al Deciphering the biology of Mycobacterium tuberculosis from the complete genome sequence. Nature. 1998;393: 537–544. doi: 10.1038/31159 9634230 10.1038/31159

[7] Dubos RJ. The effect of organic acids on mammalian tubercle bacilli. J Exp Med. The Rockefeller University Press; 1950;92: 319–332. 14778913 10.1084/jem.92.4.319 PMC2136045

[8] Marrero J, Rhee KY, Schnappinger D, Pethe K, Ehrt S. Gluconeogenic carbon flow of tricarboxylic acid cycle intermediates is critical for Mycobacterium tuberculosis to establish and maintain infection. Proc Natl Acad Sci USA. 2010;107: 9819–9824. doi: 10.1073/pnas.1000715107 20439709 10.1073/pnas.1000715107 PMC2906907

[9] Pandey AK, Sassetti CM. Mycobacterial persistence requires the utilization of host cholesterol. Proc Natl Acad Sci USA. 2008; 105: 4376–4380. doi: 10.1073/pnas.0711159105 18334639 10.1073/pnas.0711159105 PMC2393810

[10] Lee W, Vanderven BC, Fahey RJ, Russell DG. Intracellular Mycobacterium tuberculosis exploits host-derived fatty acids to limit metabolic stress. Journal of Biological Chemistry. 2013;288: 6788–6800. doi: 10.1074/jbc.M112.445056 23306194 10.1074/jbc.M112.445056 PMC3591590

[11] Singh V, Jamwal S, Jain R, Verma P, Gokhale R, Rao KVS. Mycobacterium tuberculosis-driven targeted recalibration of macrophage lipid homeostasis promotes the foamy phenotype. Cell Host Microbe. 2012;12: 669–681. doi: 10.1016/j.chom.2012.09.012 23159056 10.1016/j.chom.2012.09.012

[12] Caire-Brandli I, Papadopoulos A, Malaga W, Marais D, Canaan S, Thilo L, et al Reversible Lipid Accumulation and Associated Division Arrest of Mycobacterium avium in Lipoprotein-Induced Foamy Macrophages May Resemble Key Events during Latency and Reactivation of Tuberculosis. Infect Immun. 2014;82: 476–490. doi: 10.1128/IAI.01196-13 24478064 10.1128/IAI.01196-13 PMC3911402

[13] Daniel J, Maamar H, Deb C, Sirakova TD, Kolattukudy PE. Mycobacterium tuberculosis uses host triacylglycerol to accumulate lipid droplets and acquires a dormancy-like phenotype in lipid-loaded macrophages. PLOS Pathogens. 2011;7: e1002093 doi: 10.1371/journal.ppat.1002093 21731490 10.1371/journal.ppat.1002093 PMC3121879

[14] Mehrotra P, Jamwal SV, Saquib N, Sinha N, Siddiqui Z, Manivel V, et al Pathogenicity of Mycobacterium tuberculosis is expressed by regulating metabolic thresholds of the host macrophage. PLOS Pathogens. 2014;10: e1004265 doi: 10.1371/journal.ppat.1004265 25058590 10.1371/journal.ppat.1004265 PMC4110042

[15] Mahajan S, Dkhar HK, Chandra V, Dave S, Nanduri R, Janmeja AK, et al Mycobacterium tuberculosis Modulates Macrophage Lipid-Sensing Nuclear Receptors PPAR-γ and TR4 for Survival. The Journal of Immunology. 2012;188: 5593–5603. doi: 10.4049/jimmunol.1103038 22544925 10.4049/jimmunol.1103038

[16] Holla S, Prakhar P, Singh V, Karnam A, Mukherjee T, Mahadik K, et al MUSASHI-Mediated Expression of JMJD3, a H3K27me3 Demethylase, Is Involved in Foamy Macrophage Generation during Mycobacterial Infection. Fortune SM, editor. PLOS Pathogens. 2016;12: e1005814–25. doi: 10.1371/journal.ppat.1005814 27532872 10.1371/journal.ppat.1005814 PMC4988650

[17] Herker E, Ott M. Emerging role of lipid droplets in host/pathogen interactions. J Biol Chem. 2012;287: 2280–2287. doi: 10.1074/jbc.R111.300202 22090026 10.1074/jbc.R111.300202 PMC3268388

[18] Saka HA, Valdivia R. Emerging roles for lipid droplets in immunity and host-pathogen interactions. Annu Rev Cell Dev Biol. 2012;28: 411–437. doi: 10.1146/annurev-cellbio-092910-153958 22578141 10.1146/annurev-cellbio-092910-153958

[19] Almeida PE, Roque NR, Magalhães KG, Mattos KA, Teixeira L, Maya-Monteiro C, et al Differential TLR2 downstream signaling regulates lipid metabolism and cytokine production triggered by Mycobacterium bovis BCG infection. Biochimica et Biophysica Acta. 2014;1841: 97–107. doi: 10.1016/j.bbalip.2013.10.008 24120921 10.1016/j.bbalip.2013.10.008

[20] D'Avila H, Melo RCN, Parreira GG, Werneck-Barroso E, Castro-Faria-Neto HC, Bozza PT. Mycobacterium bovis bacillus Calmette-Guérin induces TLR2-mediated formation of lipid bodies: intracellular domains for eicosanoid synthesis in vivo. The Journal of Immunology. 2006;176: 3087–3097. doi: 10.4049/jimmunol.176.5.3087 16493068 10.4049/jimmunol.176.5.3087

[21] Flynn JL, Chan J, Triebold KJ, Dalton DK, Stewart TA, Bloom BR. An essential role for interferon gamma in resistance to Mycobacterium tuberculosis infection. J Exp Med. 1993;178: 2249–2254. 7504064 10.1084/jem.178.6.2249 PMC2191274

[22] Cooper AM, Dalton DK, Stewart TA, Griffin JP, Russell DG, Orme IM. Disseminated tuberculosis in interferon gamma gene-disrupted mice. J Exp Med. The Rockefeller University Press; 1993;178: 2243–2247. 8245795 10.1084/jem.178.6.2243 PMC2191280

[23] Casanova J-L, Abel L. Genetic dissection of immunity to mycobacteria: the human model. Annu Rev Immunol. 2002;20: 581–620. doi: 10.1146/annurev.immunol.20.081501.125851 11861613 10.1146/annurev.immunol.20.081501.125851

[24] Singh SB, Davis AS, Taylor GA, Deretic V. Human IRGM induces autophagy to eliminate intracellular mycobacteria. Science. 2006;313: 1438–1441. doi: 10.1126/science.1129577 16888103 10.1126/science.1129577

[25] MacMicking JD, North RJ, LaCourse R, Mudgett JS, Shah SK, Nathan CF. Identification of nitric oxide synthase as a protective locus against tuberculosis. Proc Natl Acad Sci. 1997;94: 5243–5248. 9144222 10.1073/pnas.94.10.5243 PMC24663

[26] MacMicking JD. Immune Control of Tuberculosis by IFN-γ-Inducible LRG-47. Science. 2003; 302: 654–659. doi: 10.1126/science.1088063 14576437 10.1126/science.1088063

[27] Kim B-H, Shenoy AR, Kumar P, Das R, Tiwari S, MacMicking JD. A family of IFN-γ-inducible 65-kD GTPases protects against bacterial infection. Science. 2011;332: 717–721. doi: 10.1126/science.1201711 21551061 10.1126/science.1201711

[28] Kimmey JM, Huynh JP, Weiss LA, Park S, Kambal A, Debnath J, et al Unique role for ATG5 in neutrophil-mediated immunopathology during M. tuberculosis infection. Nature. 2015;528: 565–569. doi: 10.1038/nature16451 26649827 10.1038/nature16451 PMC4842313

[29] Mishra BB, Rathinam VAK, Martens GW, Martinot AJ, Kornfeld H, Fitzgerald KA, et al Nitric oxide controls the immunopathology of tuberculosis by inhibiting NLRP3 inflammasome-dependent processing of IL-1β. Nat Immunol. 2013;14: 52–60. doi: 10.1038/ni.2474 23160153 10.1038/ni.2474 PMC3721324

[30] Mishra BB, Lovewell RR, Olive AJ, Zhang G, Wang W, Eugenin E, et al Nitric oxide prevents a pathogen-permissive granulocytic inflammation during tuberculosis. Nat Microbiol. Nature Publishing Group; 2017;2: 17072 doi: 10.1038/nmicrobiol.2017.72 28504669 10.1038/nmicrobiol.2017.72 PMC5461879

[31] Braverman J, Sogi KM, Benjamin D, Nomura DK, Stanley SA. HIF-1α Is an Essential Mediator of IFN-γ-Dependent Immunity to Mycobacterium tuberculosis. J Immunol. American Association of Immunologists; 2016;197: 1287–1297. doi: 10.4049/jimmunol.1600266 27430718 10.4049/jimmunol.1600266 PMC4976004

[32] Fujimoto T, Parton RG. Not Just Fat: The Structure and Function of the Lipid Droplet. Cold Spring Harbor Perspectives in Biology. 2011;3: a004838–a004838. doi: 10.1101/cshperspect.a004838 21421923 10.1101/cshperspect.a004838 PMC3039932

[33] Kimmel AR, Sztalryd C. The Perilipins: Major Cytosolic Lipid Droplet-Associated Proteins and Their Roles in Cellular Lipid Storage, Mobilization, and Systemic Homeostasis. Annu Rev Nutr. 2016;36: 471–509. doi: 10.1146/annurev-nutr-071813-105410 27431369 10.1146/annurev-nutr-071813-105410

[34] Neyrolles O, Hernandez Pando R, Pietri-Rouxel F, Fornès P, Tailleux L, Barrios Payán JA, et al Is adipose tissue a place for Mycobacterium tuberculosis persistence? Sherman D, editor. PLoS ONE. 2006;1: e43 doi: 10.1371/journal.pone.0000043 17183672 10.1371/journal.pone.0000043 PMC1762355

[35] Pol A, Gross SP, Parton RG. Biogenesis of the multifunctional lipid droplet: Lipids, proteins, and sites. J Cell Biol. 2014;204: 635–646. doi: 10.1083/jcb.201311051 24590170 10.1083/jcb.201311051 PMC3941045

[36] Wilfling F, Haas JT, Walther TC, Farese RV. Lipid droplet biogenesis. Current Opinion in Cell Biology. 2014;29: 39–45. doi: 10.1016/j.ceb.2014.03.008 24736091 10.1016/j.ceb.2014.03.008 PMC4526149

[37] Walther TC, Farese RV. Lipid droplets and cellular lipid metabolism. Annu Rev Biochem. 2012; 81: 687–714. doi: 10.1146/annurev-biochem-061009-102430 22524315 10.1146/annurev-biochem-061009-102430 PMC3767414

[38] Cao J, Zhou Y, Peng H, Huang X, Stahler S, Suri V, et al Targeting Acyl-CoA:diacylglycerol acyltransferase 1 (DGAT1) with small molecule inhibitors for the treatment of metabolic diseases. J Biol Chem. 2011;286: 41838–41851. doi: 10.1074/jbc.M111.245456 21990351 10.1074/jbc.M111.245456 PMC3308890

[39] Silverstein RL, Febbraio M. CD36, a scavenger receptor involved in immunity, metabolism, angiogenesis, and behavior. Sci Signal. 2009;2: re3–re3. doi: 10.1126/scisignal.272re3 19471024 10.1126/scisignal.272re3 PMC2811062

[40] Huang SC-C, Everts B, Ivanova Y, O’Sullivan D, Nascimento M, Smith AM, et al Cell-intrinsic lysosomal lipolysis is essential for alternative activation of macrophages. Nat Immunol. 2014;15: 846–855. doi: 10.1038/ni.2956 25086775 10.1038/ni.2956 PMC4139419

[41] Dodd CE, Pyle CJ, Glowinski R, Rajaram MVS, Schlesinger LS. CD36-Mediated Uptake of Surfactant Lipids by Human Macrophages Promotes Intracellular Growth of Mycobacterium tuberculosis. J Immunol. 2016;197: 4727–4735. doi: 10.4049/jimmunol.1600856 27913648 10.4049/jimmunol.1600856 PMC5137803

[42] Hawkes M, Li X, Crockett M, Diassiti A, Finney C, Min-Oo G, et al CD36 deficiency attenuates experimental mycobacterial infection. BMC Infect Dis. BioMed Central; 2010;10: 299 doi: 10.1186/1471-2334-10-299 20950462 10.1186/1471-2334-10-299 PMC2965149

[43] Gimm T, Wiese M, Teschemacher B, Deggerich A, Schödel J, Knaup KX, et al Hypoxia-inducible protein 2 is a novel lipid droplet protein and a specific target gene of hypoxia-inducible factor-1. FASEB J. 2010;24: 4443–4458. doi: 10.1096/fj.10-159806 20624928 10.1096/fj.10-159806

[44] Bensaad K, Favaro E, Lewis CA, Peck B, Lord S, Collins JM, et al Fatty acid uptake and lipid storage induced by HIF-1α contribute to cell growth and survival after hypoxia-reoxygenation. Cell reports. 2014;9: 349–365. doi: 10.1016/j.celrep.2014.08.056 25263561 10.1016/j.celrep.2014.08.056

[45] Peyssonnaux C, Datta V, Cramer T, Doedens A, Theodorakis EA, Gallo RL, et al HIF-1alpha expression regulates the bactericidal capacity of phagocytes. J Clin Invest. 2005;115: 1806–1815. doi: 10.1172/JCI23865 16007254 10.1172/JCI23865 PMC1159132

[46] Tannahill GM, Curtis AM, Adamik J, Palsson-McDermott EM, McGettrick AF, Goel G, et al Succinate is an inflammatory signal that induces IL-1β through HIF-1α. Nature. 2013;496: 238–242. doi: 10.1038/nature11986 23535595 10.1038/nature11986 PMC4031686

[47] Lin AE, Beasley FC, Olson J, Keller N, Shalwitz RA, Hannan TJ, et al Role of Hypoxia Inducible Factor-1α (HIF-1α) in Innate Defense against Uropathogenic Escherichia coli Infection. PLOS Pathogens. 2015;11: e1004818 doi: 10.1371/journal.ppat.1004818 25927232 10.1371/journal.ppat.1004818 PMC4415805

[48] Elks PM, Brizee S, van der Vaart M, Walmsley SR, van Eeden FJ, Renshaw SA, et al Hypoxia inducible factor signaling modulates susceptibility to mycobacterial infection via a nitric oxide dependent mechanism. Schneider DS, editor. PLOS Pathogens. 2013;9: e1003789 doi: 10.1371/journal.ppat.1003789 24367256 10.1371/journal.ppat.1003789 PMC3868520

[49] Braverman J, Stanley SA. Nitric Oxide Modulates Macrophage Responses to Mycobacterium tuberculosis Infection through Activation of HIF-1α and Repression of NF-κB. J Immunol. 2017;199: 1805–1816. doi: 10.4049/jimmunol.1700515 28754681 10.4049/jimmunol.1700515 PMC5568107

[50] DiStefano MT, Danai LV, Roth Flach RJ, Chawla A, Pedersen DJ, Guilherme A, et al The Lipid Droplet Protein Hypoxia-inducible Gene 2 Promotes Hepatic Triglyceride Deposition by Inhibiting Lipolysis. Journal of Biological Chemistry. 2015;290: 15175–15184. doi: 10.1074/jbc.M115.650184 25922078 10.1074/jbc.M115.650184 PMC4463459

[51] Chen M, Divangahi M, Gan H, Shin DSJ, Hong S, Lee DM, et al Lipid mediators in innate immunity against tuberculosis: opposing roles of PGE2 and LXA4 in the induction of macrophage death. J Exp Med. 2008;205: 2791–2801. doi: 10.1084/jem.20080767 18955568 10.1084/jem.20080767 PMC2585850

[52] Tobin DM, Vary JC, Ray JP, Walsh GS, Dunstan SJ, Bang ND, et al The lta4h locus modulates susceptibility to mycobacterial infection in zebrafish and humans. Cell. 2010;140: 717–730. doi: 10.1016/j.cell.2010.02.013 20211140 10.1016/j.cell.2010.02.013 PMC2907082

[53] Mayer-Barber KD, Andrade BB, Oland SD, Amaral EP, Barber DL, Gonzales J, et al Host-directed therapy of tuberculosis based on interleukin-1 and type I interferon crosstalk. Nature. 2014;511: 99–103. doi: 10.1038/nature13489 24990750 10.1038/nature13489 PMC4809146

[54] Muñoz-Elías EJ, McKinney JD. Mycobacterium tuberculosis isocitrate lyases 1 and 2 are jointly required for in vivo growth and virulence. Nat Med. 2005;11: 638–644. doi: 10.1038/nm1252 15895072 10.1038/nm1252 PMC1464426

[55] Nolan SJ, Romano JD, Coppens I. Host lipid droplets: An important source of lipids salvaged by the intracellular parasite Toxoplasma gondii. Billker O, editor. PLOS Pathogens. 2017;13: e1006362 doi: 10.1371/journal.ppat.1006362 28570716 10.1371/journal.ppat.1006362 PMC5469497

[56] Sassetti CM, Boyd DH, Rubin EJ. Genes required for mycobacterial growth defined by high density mutagenesis. Mol Microbiol. 2003;48: 77–84. doi: 10.1046/j.1365-2958.2003.03425.x 12657046 10.1046/j.1365-2958.2003.03425.x

[57] Cáceres N, Tapia G, Ojanguren I, Altare F, Gil O, Pinto S, et al Evolution of foamy macrophages in the pulmonary granulomas of experimental tuberculosis models. Tuberculosis. 2009;89: 175–182. doi: 10.1016/j.tube.2008.11.001 19110471 10.1016/j.tube.2008.11.001

[58] Guirado E, Mbawuike U, Keiser TL, Arcos J, Azad AK, Wang S-H, et al Characterization of Host and Microbial Determinants in Individuals with Latent Tuberculosis Infection Using a Human Granuloma Model. mBio. 2015;6: e02537–14. doi: 10.1128/mBio.02537-14 25691598 10.1128/mBio.02537-14 PMC4337582

[59] Crucet M, Wüst SJA, Spielmann P, Lüscher TF, Wenger RH, Matter CM. Hypoxia enhances lipid uptake in macrophages: role of the scavenger receptors Lox1, SRA, and CD36. Atherosclerosis. 2013;229: 110–117. doi: 10.1016/j.atherosclerosis.2013.04.034 23706521 10.1016/j.atherosclerosis.2013.04.034

[60] Bowdish DME, Sakamoto K, Kim M-J, Kroos M, Mukhopadhyay S, Leifer CA, et al MARCO, TLR2, and CD14 are required for macrophage cytokine responses to mycobacterial trehalose dimycolate and Mycobacterium tuberculosis. Ramakrishnan L, editor. PLOS Pathogens. 2009;5: e1000474 doi: 10.1371/journal.ppat.1000474 19521507 10.1371/journal.ppat.1000474 PMC2688075

[61] Gao Y, Min K, Zhang Y, Su J, Greenwood M, Gronert K. Female-Specific Downregulation of Tissue Polymorphonuclear Neutrophils Drives Impaired Regulatory T Cell and Amplified Effector T Cell Responses in Autoimmune Dry Eye Disease. J Immunol. 2015;195: 3086–3099. doi: 10.4049/jimmunol.1500610 26324767 10.4049/jimmunol.1500610 PMC4575884

[62] Moltke von J, Trinidad NJ, Moayeri M, Kintzer AF, Wang SB, van Rooijen N, et al Rapid induction of inflammatory lipid mediators by the inflammasome in vivo. Nature. 2012;490: 107–111. doi: 10.1038/nature11351 22902502 10.1038/nature11351 PMC3465483

