# Supplementary figures and images for: Lipid droplet formation in Mycobacterium tuberculosis infected macrophages requires IFN-γ/HIF-1α signaling and supports host defense
Source: PLoS Pathog. 2018 Jan 25;14(1):e1006874. doi: 10.1371/journal.ppat.1006874 (PMC5800697; doi:10.1371/journal.ppat.1006874)

Figure S1

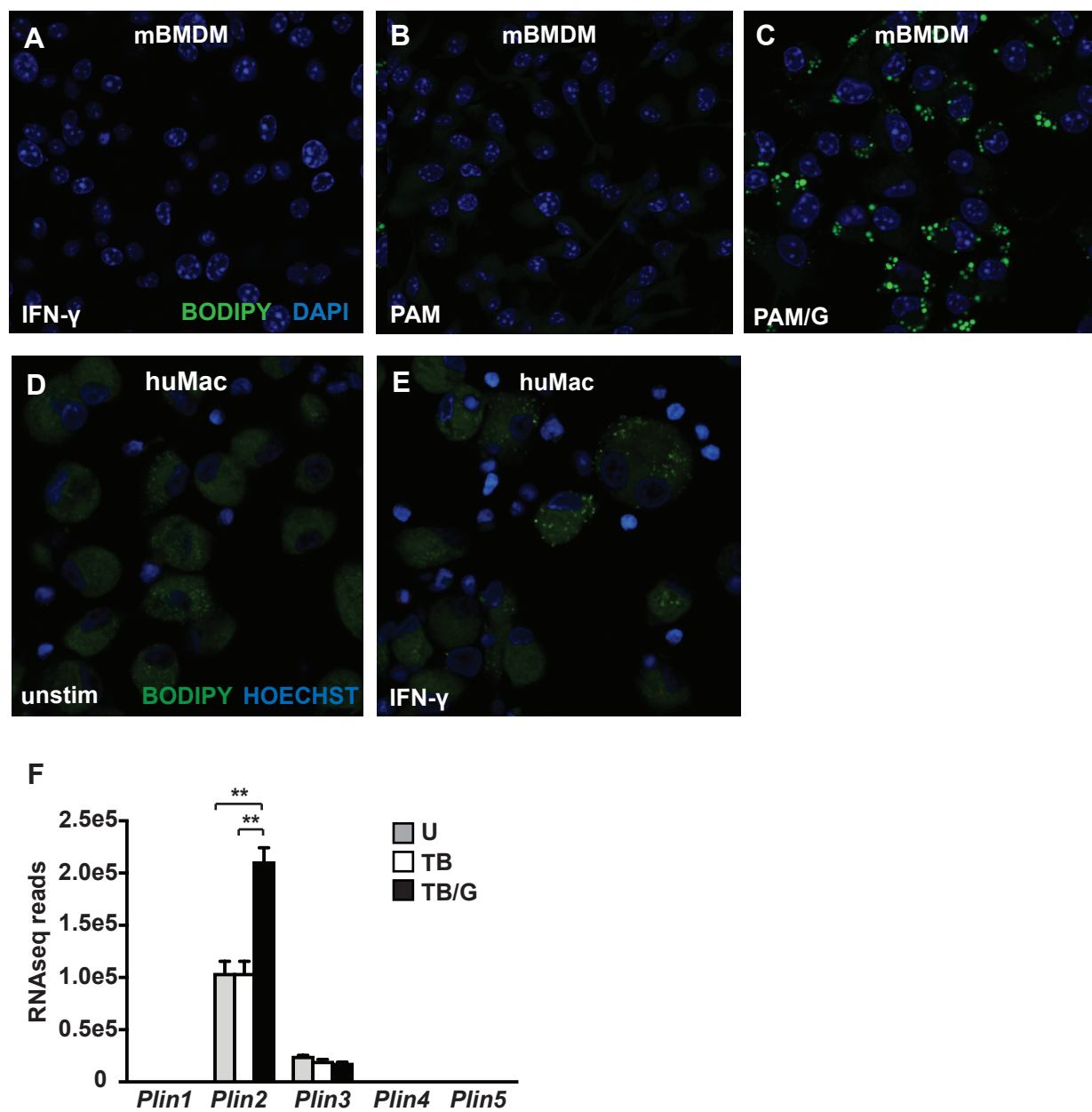

Supplement: S1 Fig — Wildtype BMDM were treated with (A) IFN-γ, (B) Pam3CSK4, or (C) IFN-γ and Pam3CSK4 for 24 hours, fixed, and imaged by confocal microscopy. Pam3CSK4 was used at 50ng/mL. Nuclei were stained with DAPI and neutral lipids were stained with BODIPY 493/503. (D,E) Primary human monocyte derived macrophages were (D) unstimulated or (E) IFN-γ treated for 24 hours, fixed, and imaged by confocal microscopy. Nuclei were stained with Hoechst 33342 and neutral lipids with BODIPY 493/503. (F) RNA-seq data showing transcript levels of members of the PLIN/PAT protein family in wildtype BMDM 24 hours post-infection in uninfected [U], M. tuberculosis infected [TB], or IFN-γ activated and M. tuberculosis infected [TB/G] BMDM. Figures are representative of a minimum of three experiments with the exception of human macrophage experiments which were performed in duplicate. Error bars are standard deviation. **p<0.01 by unpaired t-test. (PDF) [file ppat.1006874.s001.pdf]

Figure S2

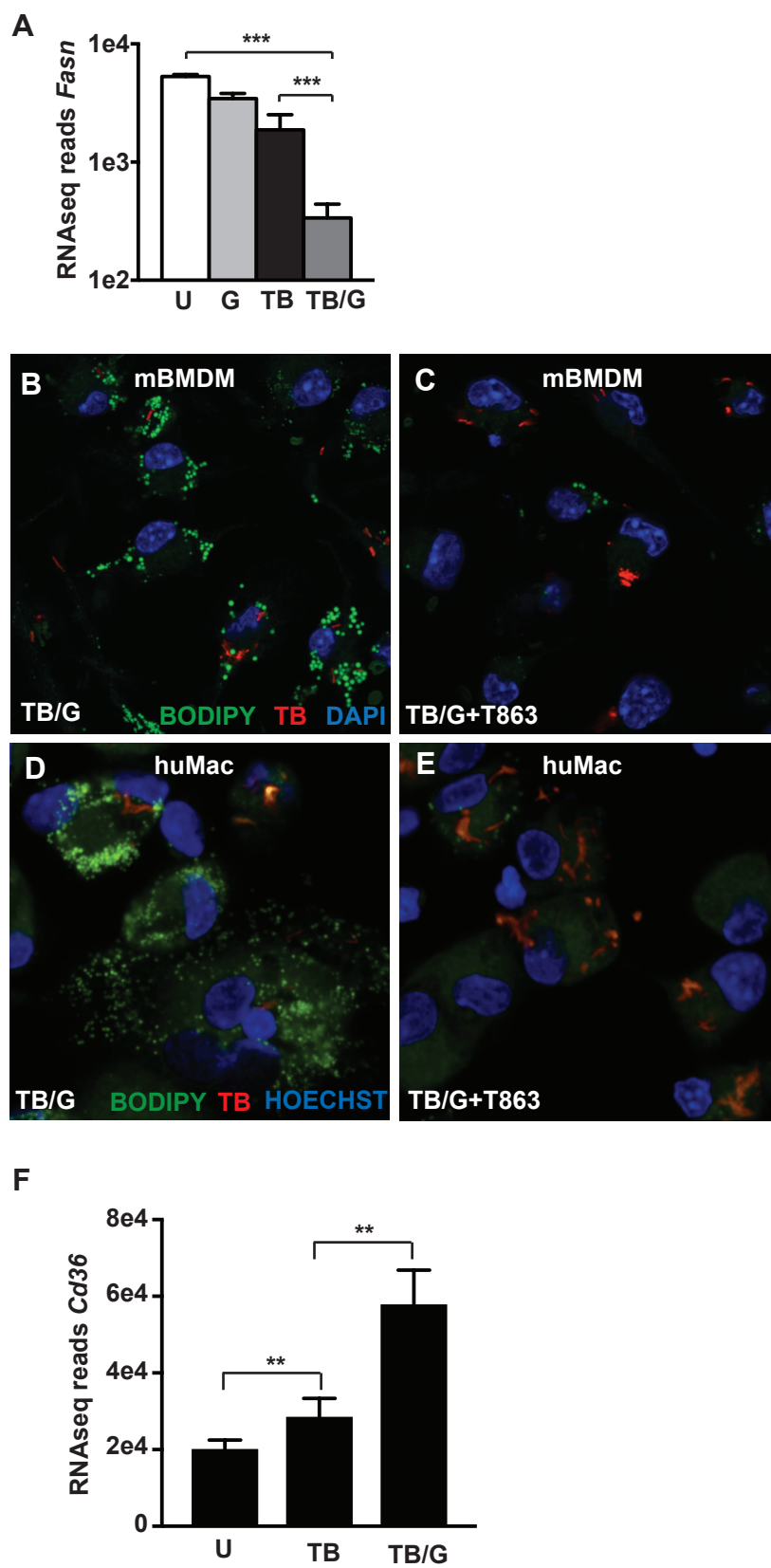

Supplement: S2 Fig — (A) RNA-seq data showing transcript levels of Fasn in wildtype BMDM 24 hours post-infection in uninfected [U], IFN-γ activated [G], M. tuberculosis infected [TB], or IFN-γ activated and M. tuberculosis infected [TB/G] BMDM. (B,C) IFN-γ activated BMDM were infected with M. tuberculosis 635-Turbo and the DGAT1 inhibitor T863 was added after the 4 hour phagocytosis period (C). BMDM were imaged 3 days post-infection by confocal microscopy. Nuclei were stained with DAPI and neutral lipids were stained with BODIPY 493/503. (D,E) IFN-γ activated primary human monocyte derived macrophages were infected with M. tuberculosis 635-Turbo and the DGAT1 inhibitor T863 was added after the 4 hour phagocytosis period (E), and were imaged 1 day post-infection by confocal microscopy. Nuclei were stained with Hoechst 33342 and neutral lipids were stained with BODIPY 493/503. (F) RNA-seq data showing transcript levels of Cd36 in wildtype BMDM 24 hours post-infection. Figures are representative of a minimum of three independent experiments, with the exception of human macrophage experiments which were performed in duplicate. Error bars are standard deviation. **p<0.01, ***p < .001 by unpaired t-test. (PDF) [file ppat.1006874.s002.pdf]

Figure S3

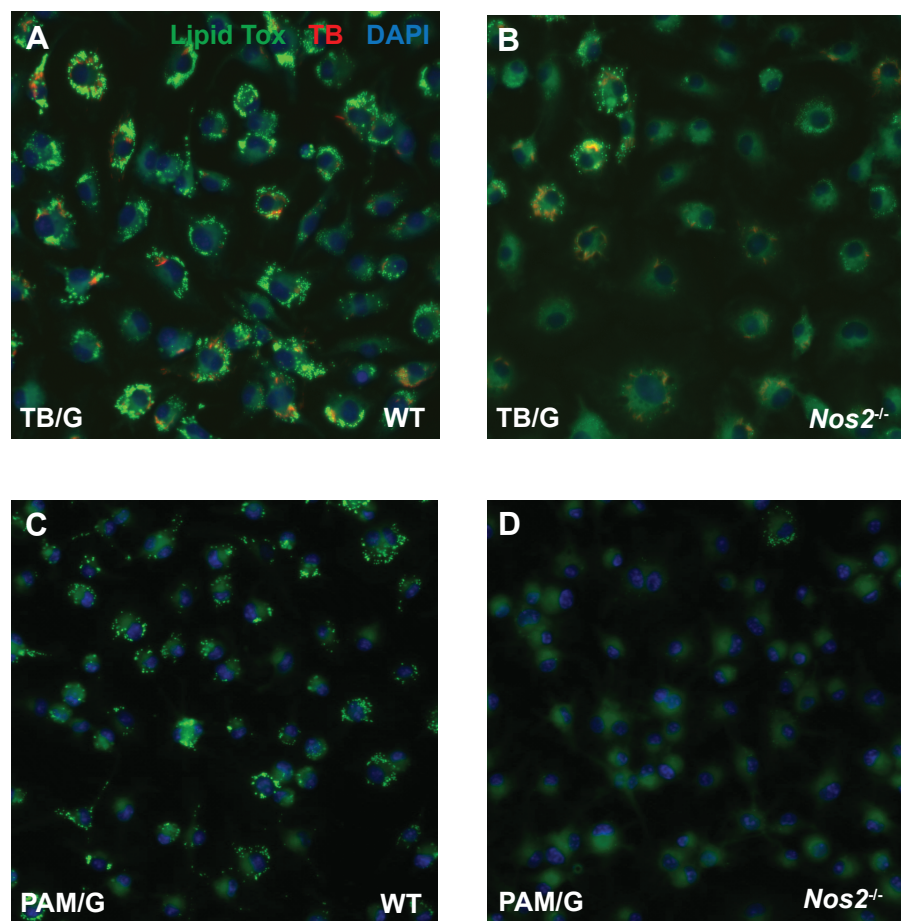

Supplement: S3 Fig — (A,B) Wildtype and Nos2-/- BMDM were activated with IFN-γ and infected with M. tuberculosis 635-Turbo. Nuclei were stained with DAPI and neutral lipids were stained with LipidTox Green. (C,D) Wildtype and Nos2-/- BMDM were treated with Pam3CSK4 and IFN-γ. Nuclei were stained with DAPI and neutral lipids were stained with LipidTox Green. Figures are representative of a minimum of three independent experiments. (PDF) [file ppat.1006874.s003.pdf]

Figure S4

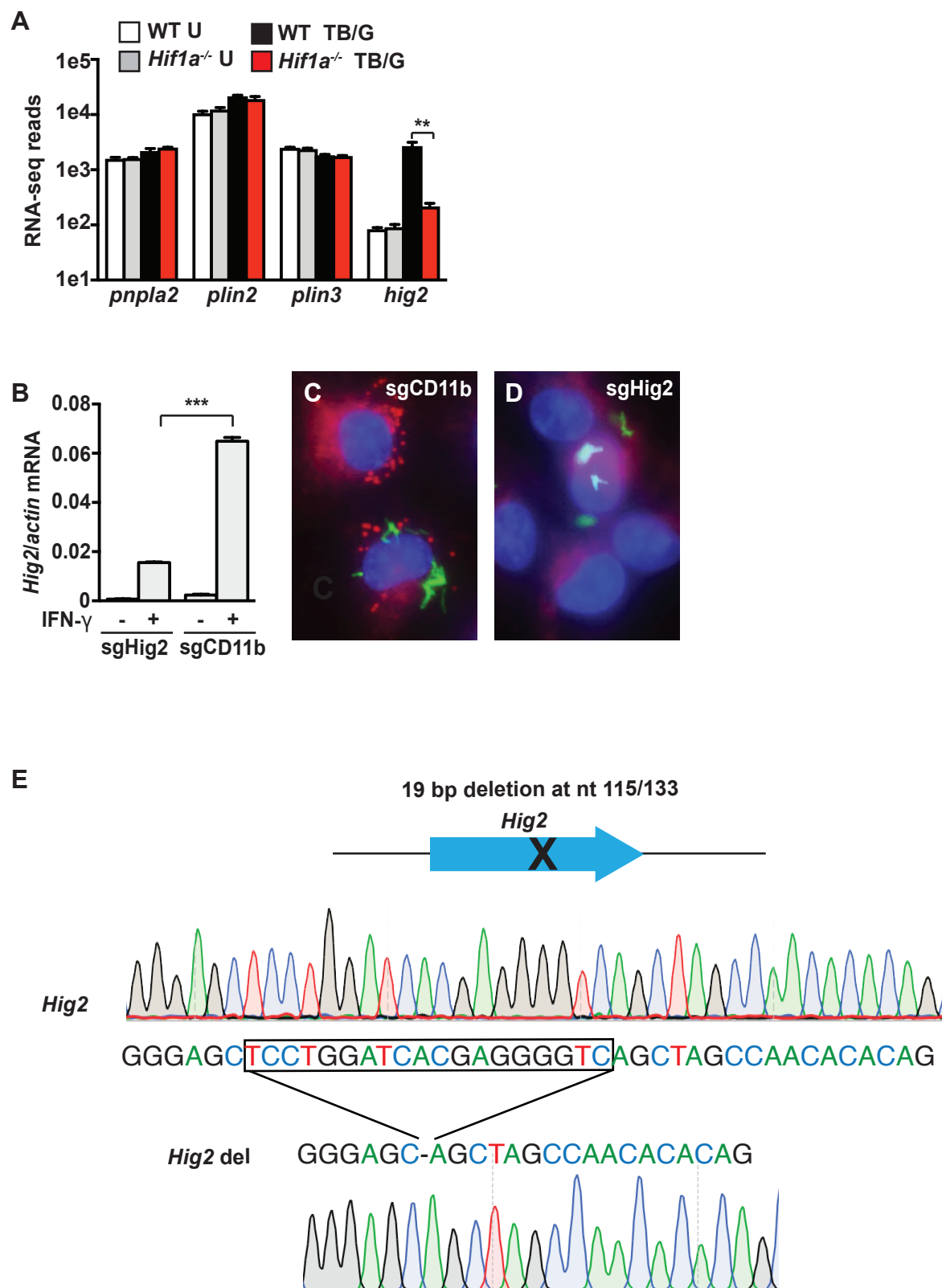

Supplement: S4 Fig — (A) RNA-seq data showing expression levels of LD associated genes in wildtype and Hif1a-/- BMDM uninfected [U] and IFN-γ activated and M. tuberculosis infected [TB/G]. Timepoint is 24 hours post-infection. (B) Cas9 transgenic BMDM stably expressing single guides targeting Hig2 or Cd11b were infected with M. tuberculosis-GFP with and without IFN-γ activation. RNA was isolated and qPCR data is shown for expression of Hig2 normalized to actin (Actb). (C,D) Cas9 transgenic BMDM stably expressing single guides targeting Hig2 (C) or Cd11b (D) were activated with IFN-γ and infected with M. tuberculosis-GFP. 1 day post-infection, BMDM were fixed, nuclei were stained with DAPI, LDs were stained with LipidTox Red, and widefield microscopy was performed. (E) A Hig2-/- mouse was generated using CRISPR/Cas9 and one sgRNA targeted to the Hig2 exon that induced a 19 nucleotide deletion and frameshift mutation. Sequence and chromatogram of the wildtype Hig2 and the Hig2 deletion allele are shown. Error bars are standard deviation, **p<0.01, ***p<0.001 by unpaired t-test. (PDF) [file ppat.1006874.s004.pdf]

Figure S5

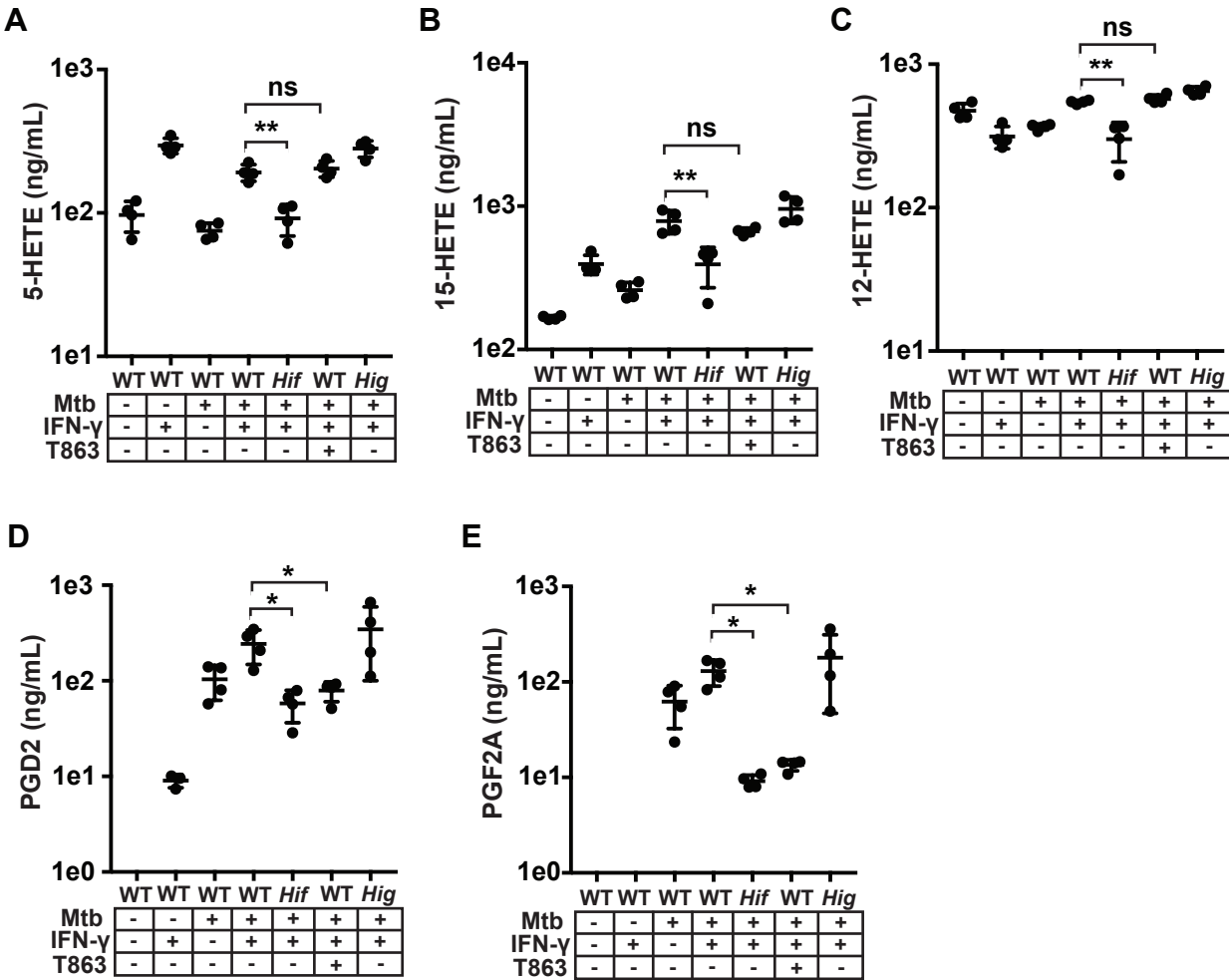

Supplement: S5 Fig — (A-E) LC-MS/MS based eicosanoid profiling was performed on BMDM supernatants 48 hours post-infection with M. tuberculosis in wildtype [WT], Hif1a-/- [Hif], and Hig2-/- [Hig] BMDM. IFN-γ and DGAT1 inhibitor T863 treatment was added as indicated. Data is shown for the following eicosanoids: (A) 5-HETE, (B) 15-HETE, (C) 12-HETE, (D) PGD2, and (E) PGF2A. Eicosanoid profiling was performed once in quadruplicate. Error bars are standard deviation, *p<0.05, **p<0.01 by unpaired t-test. (PDF) [file ppat.1006874.s005.pdf]

Figure S6

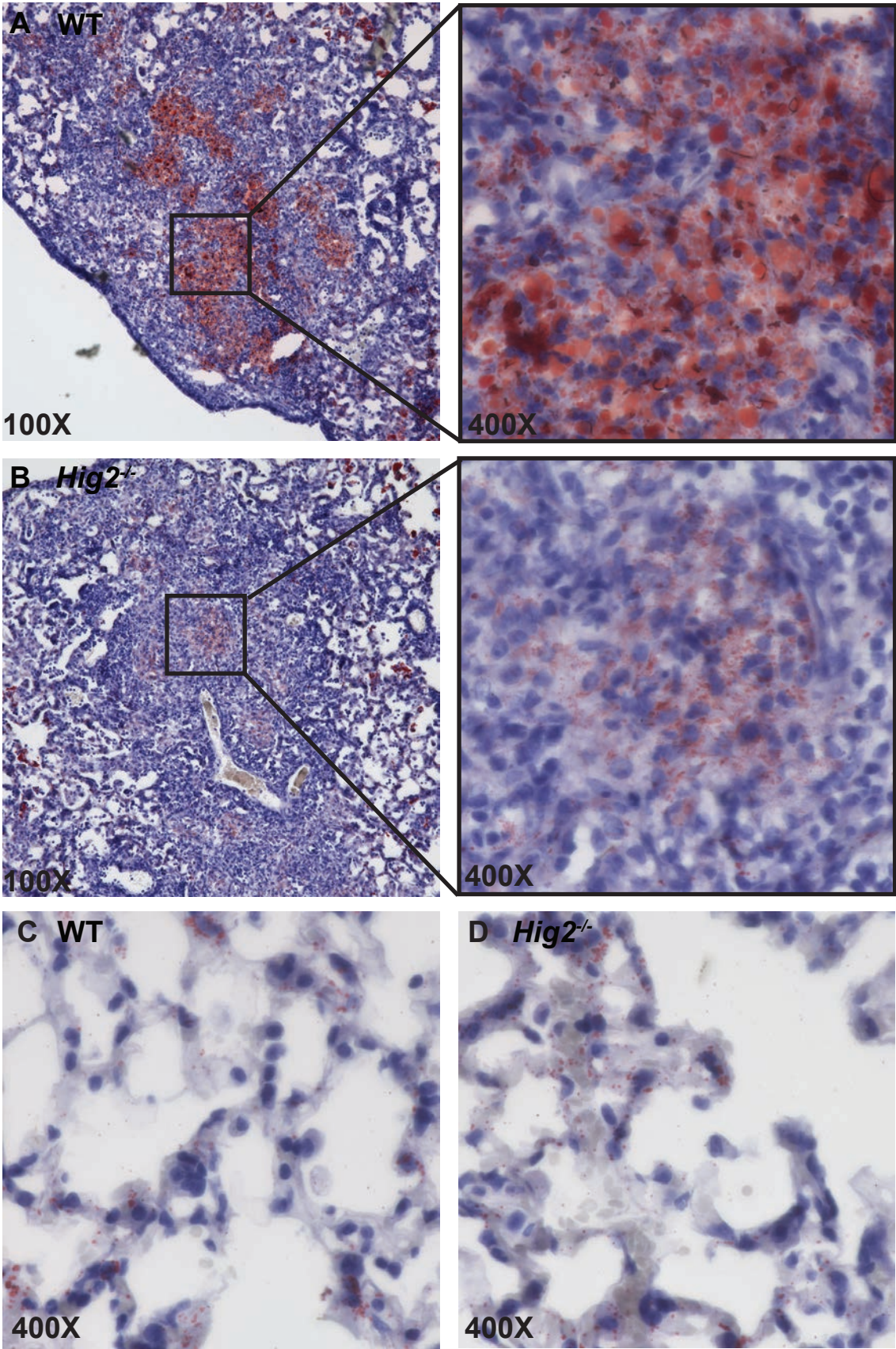

Supplement: S6 Fig — Mice were infected with ~200 CFU of M. tuberculosis Erdman via the aerosol route and lungs were collected for histological analysis of LD formation by Oil Red O (ORO) staining. Sections were counterstained with hematoxylin. (A) ORO accumulation in wildtype lung lesions as in Fig 7E and 7F, but with higher resolution and digital zoom. (B) ORO accumulation in Hig2-/- lung lesions as in Fig 7I and 7J. Higher resolution images show that in Hig2-/- lesions, the LDs that are present are smaller. (C,D) ORO staining LDs were observed in the epithelium in non-lesion areas in wildtype (C) and Hig2-/- (D) lungs, with no apparent difference between genotypes. (PDF) [file ppat.1006874.s006.pdf]
