# Supplementary material for: Lipid droplet formation in Mycobacterium tuberculosis infected macrophages requires IFN-γ/HIF-1α signaling and supports host defense
Source: PLoS Pathog. 2018 Jan 25;14(1):e1006874. doi: 10.1371/journal.ppat.1006874 (PMC5800697; doi:10.1371/journal.ppat.1006874)
Supplement: S1 Table — Lipidomic quantification of cellular lipids from wildtype and Hif1a-/- BMDM that were untreated (U), IFN-γ activated (G), M. tuberculosis infected (TB), or IFN-γ activated and M. tuberculosis infected (TB/G). Samples were prepared 24 hours post-infection. Values listed are the average uM concentration from 5 biological replicates, with standard deviation listed in parentheses. Lipids profiled are cholesteryl ester (CE), triacylglycerol (TAG), diacylglycerol (DAG), free fatty acid (FFA), phosphatidylcholine (PC), phosphatidylethanolamine (PE), phosphatidylinositol (PI), lysophosphatidylcholine (LPC), lysophosphatidylethanolamine (LPE), sphingomyelin (SM), ceramide (CER), hexosylceramide (HCER), lactosylceramide (LCER), dihydroceramide (DCER). (DOCX) [file ppat.1006874.s007.docx]

|  |  | Wildtype BMDM | | | | *Hif1a^-/-^* BMDM | | | |
| --- | --- | --- | --- | --- | --- | --- | --- | --- | --- |
|  |  | U | G | TB | TB/G | U | G | TB | TB/G |
| Group | Lipid Class |  |  |  |  |  |  |  |  |
| Neutral lipids | CE | 11.45 (1.82) | 19.49 (1.55) | 52.55 (5.07) | 109.78 (10.99) | 9.78 (1.62) | 18.58 (2.81) | 62.13 (5.01) | 87.93 (6.14) |
|  | TAG | 103.10  (6.62) | 151.72  (15.04) | 202.13 (12.90) | 232.28  (14.34) | 105.83 (3.73) | 148.73 (13.47) | 189.98 (10.91) | 214.14 (16.40) |
|  | DAG | 94.62 (6.10) | 105.82 (11.58) | 119.59 (31.00) | 121.65  (15.91) | 92.87 (13.54) | 137.60 (19.18) | 157.79 (13.37) | 180.20  (6.15) |
|  | FFA | 1745.97(127.95) | 1965.86  (283.41) | 2314.32  (593.46) | 1800.84  (147.23) | 1918.25  (154.69) | 2229.77  (244.40) | 2392.23  (110.14) | 2442.65  (191.56) |
| Phospholipids | PC | 3065.01 (144.74) | 3725.82 (320.12) | 2845.48 (200.49) | 3828.60 (149.85) | 3144.33 (195.62) | 3845.48 (243.64) | 3209.83 (131.31) | 3870.89 (214.35) |
|  | PE | 5200.21 (370.38) | 5541.51 (659.64) | 4572.45 (496.24) | 5648.84 (270.18) | 5066.94 (313.60) | 5371.54 (503.11) | 4753.26 (364.11) | 5491.53 (423.75) |
|  | PI | 220.21 (16.92) | 272.97 (48.89) | 237.46 (44.42) | 285.23 (19.01) | 223.89 (31.85) | 260.76 (25.80) | 252.40 (13.25) | 307.72 (40.78) |
|  | LPC | 23.93 (4.05) | 40.80 (3.56) | 27.12 (2.56) | 37.00 (2.08) | 29.99 (2.37) | 49.15 (4.44) | 43.12 (3.30) | 60.67 (5.98) |
|  | LPE | 11.91 (2.38) | 17.14 (2.38) | 17.43 (1.63) | 19.46 (1.09) | 13.07 (1.00) | 21.42 (1.57) | 22.83 (1.51) | 26.79 (1.87) |
| Sphingolipids | SM | 1883.93 (99.13) | 2053.65 (201.36) | 1984.41 (74.16) | 2072.41 (49.39) | 1869.92 (75.13) | 1940.26 (128.39) | 2039.53 (56.32) | 2303.04 (157.61) |
|  | CER | 121.40 (12.98) | 169.33 (22.03) | 119.28 (10.30) | 111.49 (9.10) | 112.71 (7.64) | 157.91 (13.72) | 114.71 (3.56) | 112.70 (13.02) |
|  | HCER | 77.76 (8.81) | 154.82 (25.94) | 109.81 (3.93) | 106.62 (5.73) | 84.98 (5.07) | 156.68 (18.78) | 100.16 (11.25) | 155.18 (7.77) |
|  | LCER | 2.56 (1.26) | 4.10 (1.02) | 6.12 (1.35) | 4.16 (0.39) | 2.84 (1.13) | 4.23 (0.95) | 6.05 (0.60) | 5.51 (0.68) |
|  | DCER | 13.71 (1.93) | 20.34 (3.35) | 15.37 (2.48) | 15.99 (1.54) | 14.30 (2.27) | 20.13 (3.63) | 14.07 (1.53) | 16.14 (1.59) |
