# Supplementary material for: Lipid droplet formation in Mycobacterium tuberculosis infected macrophages requires IFN-γ/HIF-1α signaling and supports host defense
Source: PLoS Pathog. 2018 Jan 25;14(1):e1006874. doi: 10.1371/journal.ppat.1006874 (PMC5800697; doi:10.1371/journal.ppat.1006874)
Supplement: S2 Table — Lipidomic quantification of eicosanoids from untreated wildtype BMDM (WT U), IFN-γ activated wildtype BMDM (WT G), M. tuberculosis infected wildtype BMDM (WT TB), IFN-γ activated and M. tuberculosis infected wildtype BMDM (WT TB/G), IFN-γ activated and M. tuberculosis infected Hif1a-/- BMDM (Hif TB/G), IFN-γ activated and M. tuberculosis infected wildtype BMDM treated with T863 (WT T863), and IFN-γ activated and M. tuberculosis infected Hig2-/- BMDM (Hig TB/G). Values listed are the average concentrations in pg/ml from 4 biological replicates, with standard deviations listed in parentheses. For each eicosanoid listed, the value in the top row represents the concentration at 48 hours post-infection and value in the bottom row represents the concentration at 72 hours post-infection (following a media change at 48 hours post-infection). Eicosanoids listed: arachidonic acid (AA); 5-, 12-, and 15-hydroxyeicosatetraenoic acid (5-,12-, 15-HETE); prostaglandin E2 (PGE2); prostaglandin D2 (PGD2); prostaglandin F2alpha (PGF2a); lipoxin A4 (LXA4); lipoxin B4 (LXB4); thromboxane B2 (TXB2); docosahexaenoic acid (DHA); 4-, 7-, 14-, and 17-hydroxy docosahexanoic acid (4-, 7-, 14-, 17-HDHA); eicosapentaenoic acid (EPA); 12-, 15-, and 18-hydroxyeicosapentaenoic acid (12-, 15-, 18-HEPE); 13-hydroxyoctadecadienoic acid (13-HODE). *N.D. = not detected. (DOCX) [file ppat.1006874.s008.docx]

|  | WT U | WT G | WT TB | WT TB/G | *Hif* TB/G | WT T863 | *Hig* TB/G |
| --- | --- | --- | --- | --- | --- | --- | --- |
| AA | 908.5  (43.8) | 3,079.3  (391.6) | 483.5  (42.2) | 523.4  (13.1) | 405.2  (122.8) | 507.3  (76.7) | 721.7  (97.1) |
|  | 1,157.9  (125.1) | 5,088.4  (692.4) | 1,513.9  (124.9) | 3,898.0  (679.0) | 1,793.8  (278.3) | 3,865.9  (531.4) | 5,749.7  (1113.0) |
| 5-HETE | 96.9  (23.4) | 295.7  (36.5) | 75.1  (9.9) | 192.0  (25.5) | 91.8  (22.7) | 204.0  (26.6) | 281.3  (36.8) |
|  | 136.1  (18.5) | 453.4  (62.1) | 328.6  (31.7) | 636.9  (89.7) | 425.6  (70.0) | 659.1  (57.8) | 812.8  (139.2) |
| 12-HETE | 473.2  (58.3) | 312.7  (55.0) | 366.0  (18.3) | 545.2  (15.1) | 300.5  (92.1) | 573.0  (38.6) | 649.9  (45.0) |
|  | 1,905.1  (146.7) | 1,456.7  (177.7) | 1,982.7  (176.9) | 3,206.1  (392.0) | 2,166.7  (312.8) | 2,857.1  (314.1) | 3,917.6  (978.4) |
| 15-HETE | 166.9  (5.2) | 394.2  (60.5) | 259.7  (33.8) | 786.5  (143.6) | 392.3  (123.1) | 666.3  (38.6) | 956.7  (200.9) |
|  | 438.5  (458.4) | 414.9  (49.0) | 486.5  (59.6) | 608.8  (25.1) | 545.7  (56.7) | 635.1  (62.6) | 2,588.4  (3934.6) |
| PGE2 | 1.2  (0.7) | 1.6  (0.6) | 16.8  (4.1) | 54.6  (28.7) | 18.7  (7.6) | 22.6  (4.7) | 78.9  (38.2) |
|  | N.D. | N.D. | 3.9  (0.3) | 5.0  (2.0) | 4.5  (0.4) | 2.9  (0.3) | 7.7  (3.05) |
| PGD2 | N.D. | 6.8  (4.7) | 103.8  (41.2) | 243.9  (95.5) | 58.1  (21.6) | 79.0  (18.7) | 346.4  (246.1) |
|  | N.D. | N.D. | 28.8  (2.0) | 21.0  (6.1) | 25.5  (2.7) | 18.1  (1.7) | 26.2  (6.4) |
| PGF2a | N.D. | N.D. | 62.0  (29.6) | 129.7  (39.3) | 9.1  (1.5) | 13.5  (1.8) | 179.8  (132.8) |
|  | 8.2  (4.2) | N.D. | 9.5  (1.2) | 18.6  (4.8) | 0.1  (0.2) | 9.5  (2.5) | 15.8  (5.9) |
| LXA4 | 767.3  (771.1) | 800.9  (647.3) | 848.3  (610.6) | 707.0  (526.6) | 302.8  (92.5) | 545.7  (65.4) | 729.6  (314.1) |
|  | 245.6  (34.4) | 155.2  (25.7) | 308.9  (19.0) | 301.5  (90.2) | 313.6  (58.8) | 311.7  (84.7) | 380.8  (189.6) |
| LXB4 | N.D. | N.D. | 1,311.6  (574.6) | 4,545.4  (1682.7) | 925.7  (495.7) | 1,278.7  (198.0) | 7,431.6  (3716.2) |
|  | N.D. | N.D. | 327.6  (90.8) | 454.6  (274.7) | 566.7  (70.5) | 507.2  (176.8) | 491.9  (174.4) |
| TXB2 | 253.3  (402.0) | 52.5  (21.8) | 398.9  (660.3) | 590.5  (426.1) | 1,000.2  (327.5) | 1,475.8  (178.4) | 1,136.7  (1023.4) |
|  | 589.9  (54.1) | 660.4  (72.9) | 665.7  (36.5) | 749.3  (57.9) | 824.1  (81.6) | 829.0  (92) | 816.4  (140.6) |
| DHA | 1,871.1  (104.8) | 4,006.6  (141.2) | 854.4  (36.3) | 1,461.6  (150.6) | 952.5  (267.3) | 1,334.4  (72.1) | 2,185.6  (130.7) |
|  | 2,239.7  (191.8) | 4,584.5  (652.5) | 2,523.2  (156.1) | 4,898.9  (312.1) | 3,053.2  (308.6) | 4,657.2  (231.5) | 6,007.8  (535.3) |
| 4-HDHA | 162.7  (16.7) | 355.3  (83.5) | 71.1  (49.7) | 180.7  (16.7) | 126.2  (38.4) | 230.1  (45.5) | 250.8  (9.0) |
|  | 144.3  (3.1) | 413.2  (112.3) | 241.0  (18.1) | 432.4  (77.0) | 327.5  (70.4) | 428.6  (19.9) | 492.7  (116.1) |
| 7-HDHA | 156.2  (34.6) | 221.4  (14.8) | 162.9  (83.4) | 213.3  (47.5) | 151.8  (52.1) | 281.8  (66.0) | 231.5  (26.5) |
|  | 139.8  (25.8) | 169.1  (39.3) | 223.2  (27.0) | 235.8  (26.3) | 223.3  (56.8) | 238.7  (68.0) | 231.0  (91.7) |
| 14-HDHA | 633.3  (90.4) | 640.1  (117.8) | 552.2  (58.7) | 922.6  (51.4) | 815.2  (312.1) | 1,167.8  (255.0) | 1,172.3  (134.1) |
|  | 1,073.9  (173.5) | 850.0  (223.2) | 1,424.4  (337.9) | 1,932.2  (340.5) | 1,286.9  (255.3) | 1,555.6  (258.7) | 2,216.7  (979.8) |
| 17-HDHA | 778.3  (197.7) | 1,406.8  (118.1) | 700.6  (105.3) | 1,195.0  (371.6) | 1,126.0  (339.1) | 1,766.3  (179.4) | 1,397.0  (244.9) |
|  | 1,107.7  (373.8) | 1,225.7  (351.0) | 1,510.1  (229.4) | 1,501.8  (414.2) | 1,741.2  (544.7) | 2,031.6  (172.4) | 3,051.6  (2558.3) |
| EPA | 539.8  (102.5) | 2,225.4  (587.4) | 266.9  (30.9) | 633.0  (96.3) | 243.0  (149.0) | 486.0  (178.2) | 1,010.8  (353.5) |
|  | 440.9  (30.3) | 2,128.6  (93.2) | 715.7  (79.4) | 2,848.5  (388.5) | 1,070.2  (201.6) | 4,518.5  (1783.3) | 4,099.7  (579.9) |
| 12-HEPE | 162.0  (33.1) | 54.3  (1.7) | 165.0  (5.9) | 207.6  (16.4) | 144.1  (46.6) | 205.6  (49.9) | 208.3  (17.7) |
|  | 501.9  (38.8) | 259.2  (17.3) | 566.8  (38.7) | 582.8  (23.6) | 474.5  (8.7) | 555.9  (20.2) | 725.8  (91.0) |
| 15-HEPE | 94.3  (10.8) | 115.4  (17.3) | 189.2  (8.6) | 314.6  (45.3) | 201.1  (68.9) | 298.0  (45.7) | 351.3  (47.4) |
|  | 155.6  (36.6) | 163.3  (31.2) | 288.8  (48.5) | 251.5  (6.1) | 233.0  (44.0) | 278.4  (16.7) | 419.8  (318.6) |
| 18-HEPE | 170.1  (47.4) | 184.7  (19.8) | 300.9  (118.1) | 267.3  (19.1) | 170.4  (146.2) | 14.8  (4.34) | 296.4  (88.2) |
|  | 6.6  (1.4) | 2.4  (0.6) | 3.2  (0.46) | 1.8  (0.7) | 1.6  (0.3) | 1.2  (0.3) | 0.9  (0.1) |
| 13-HODE | 723.7  (80.0) | 1,084.2  (173.3) | 746.3  (158.1) | 1,108.1  (173.6) | 675.4  (225.2) | 1,059.9  (135.1) | 1,167.6  (314.3) |
|  | 715.3  (306.1) | 704.4  (101.9) | 690.7  (72.1) | 725.6  (82.8) | 691.5  (105.1) | 824.1  (59.1) | 1,925.4  (2367.1) |
